# Condensed-Phase Molecular Representation to Link Structure and Thermodynamics in Molecular Dynamics

**DOI:** 10.1021/acs.jctc.3c00201

**Published:** 2023-07-03

**Authors:** Bernadette Mohr, Diego van der Mast, Tristan Bereau

**Affiliations:** † Van ’t Hoff Institute for Molecular Sciences and Informatics Institute, University of Amsterdam, Amsterdam 1098 XH, The Netherlands; ‡ Max Planck Institute for Polymer Research, Mainz 55128, Germany

## Abstract

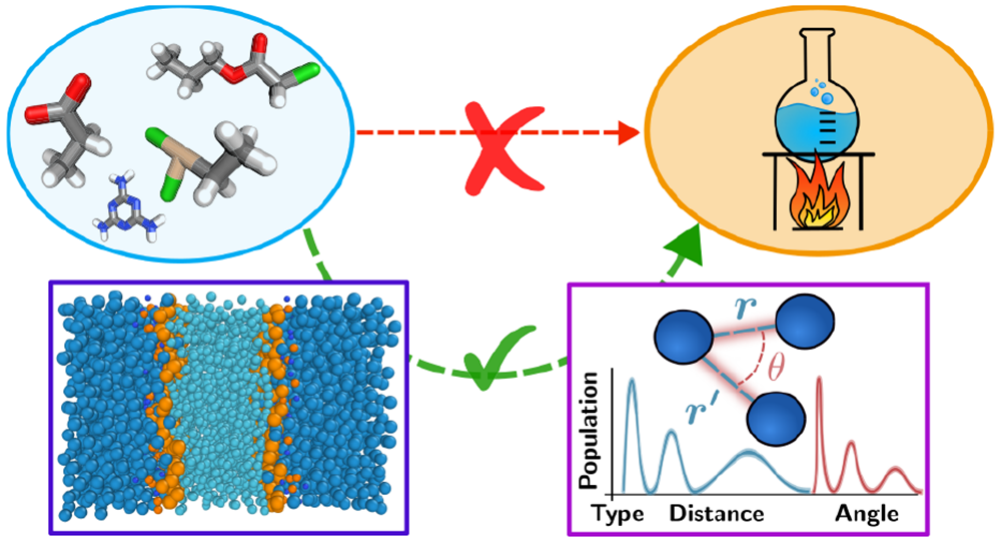

Molecular design requires systematic and broadly applicable methods to extract structure–property relationships. The focus of this study is on learning thermodynamic properties from molecular-liquid simulations. The methodology relies on an atomic representation originally developed for electronic properties: the Spectrum of London and Axilrod–Teller–Muto representation (SLATM). SLATM’s expansion in one-, two-, and three-body interactions makes it amenable to probing structural ordering in molecular liquids. We show that such representation encodes enough critical information to permit the learning of thermodynamic properties via linear methods. We demonstrate our approach on the preferential insertion of small solute molecules toward cardiolipin membranes and monitor selectivity against a similar lipid. Our analysis reveals simple, interpretable relationships between two- and three-body interactions and selectivity, identifies key interactions to build optimal prototypical solutes, and charts a two-dimensional projection that displays clearly separated basins. The methodology is generally applicable to a variety of thermodynamic properties.

## 1 Introduction

Computational molecular design is rapidly becoming one of the most exciting fields of our time thanks to its impressive developments and broad applicability.^1−6^ The idea is simple: identify molecules or materials with desirable properties. In practice, solving the underlying inverse design problem remains challenging, requiring extensive computational resources combined with an approach that exploits the underlying physics and chemistry at hand. Electronic properties have spearheaded the movement: quantum-mechanical (QM) calculations (e.g., density-functional theory) over large numbers of molecules have been successfully used in the context of machine learning (ML) to predict various properties with increasing accuracy and generalization.^7^ In no small part is this success due to the development of molecular representations: they exploit physical laws (e.g., *r*^–1^ scaling for Coulombic interactions) and account for symmetries via invariances.^8,9^ In the present study, we focus on thermodynamic properties, in particular in the context of condensed-phase liquids.

The same principles should hold when moving from electronic to thermodynamic properties: molecular representations form the basic ingredients to describe structural features, and any physical prior will help learning performance. While electronic properties typically focus on single molecules in the gas, our consideration of thermodynamic properties brings two specificities:

(1) Thermodynamics underlines the role of conformational entropy. Beyond a static structure, the diversity of conformations heavily impacts the energetics, calling for phase-space (Boltzmann) averaging.

(2) The condensed phase involves a molecule embedded in a dense environment, highlighting the balance of covalent and noncovalent interactions.

The question addressed by this study is how to efficiently learn structure–property relationships from (bio)molecular simulations of thermodynamic properties.

We take clues from the field of glassy dynamics. Impressive ML developments have been made to establish new insight into the relevant structure–dynamics relationships.^10,11^ These remarkable strides have required large and complex deep neural networks. However, significantly smaller ML models can be used when exploiting relevant physics: representations that focus on the local as well as neighboring structure.^12^ The representations often consist of structural order parameters, in particular radial and angular structure functions. While the radial (i.e., two-body) component measures the density of particles, akin to a radial distribution function (RDF), the angular terms are inspired by bond-orientational order parameters, that is, three-body interactions.^13^ The description of molecular systems in terms of an increasing number of interacting particles is called a body-order expansion.^8^

**Figure 1:**
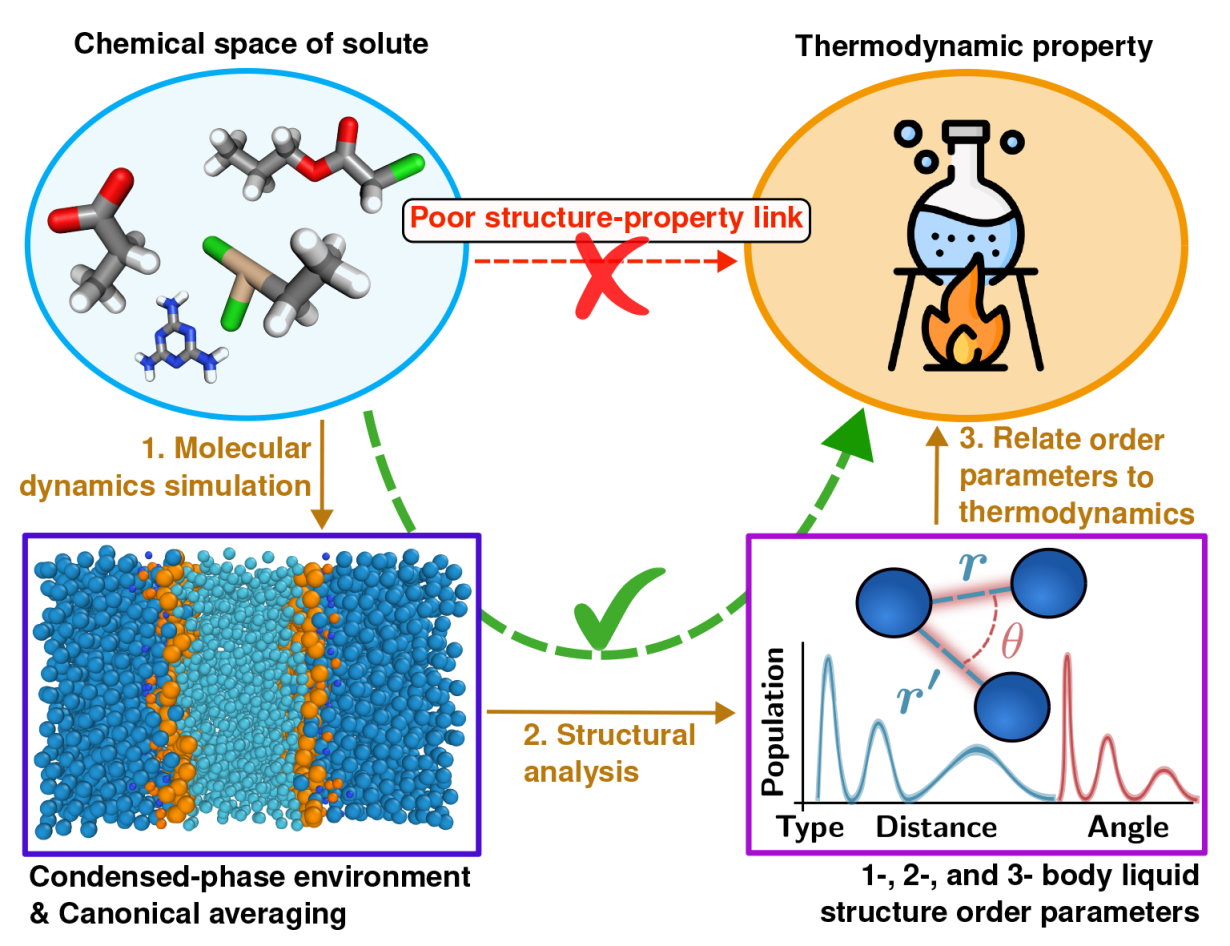
This study considers the identification of structure–property relationships between a solute molecule and a target thermodynamic property. Rather than directly learning the relationship, we propose a three-step process: (1) molecular dynamics simulations of the solute in its condensed-phase environment; (2) structural analysis of the liquid structure; and (3) relating structural order parameters and thermodynamic properties. The learning procedure thus relies on features that incorporate relevant physics, including Boltzmann phase-space averaging, liquid environment, and collective effects. Our methodology is able to identify complex structure–property relationships, even with a simple linear model. Icon from flaticon.com.

In this work, we adapt the idea of structural body-order interactions to learn thermodynamic properties in molecular simulations. We propose to start from an atomic representation originally developed for the machine learning of electronic properties: the Spectrum of London Axilrod–Teller–Muto representation (SLATM).^14,15^ SLATM provides a body-order expansion through a histogram of one-, two-, and three-body atomic contributions. Moreover, it does not distinguish between covalent and noncovalent interactions, making it well suited for a condensed phase. Finally, we extend its role to a Boltzmann ensemble by averaging over snapshots of a molecular dynamics (MD) trajectory.^16−18^Figure 1 sketches our approach: from chemical-space compound screening to thermodynamic properties via the structural analysis of MD simulations. When establishing structure–property relationships, we expect the structural order parameters to encode critical information that will ease the learning process.

**Figure 2:**
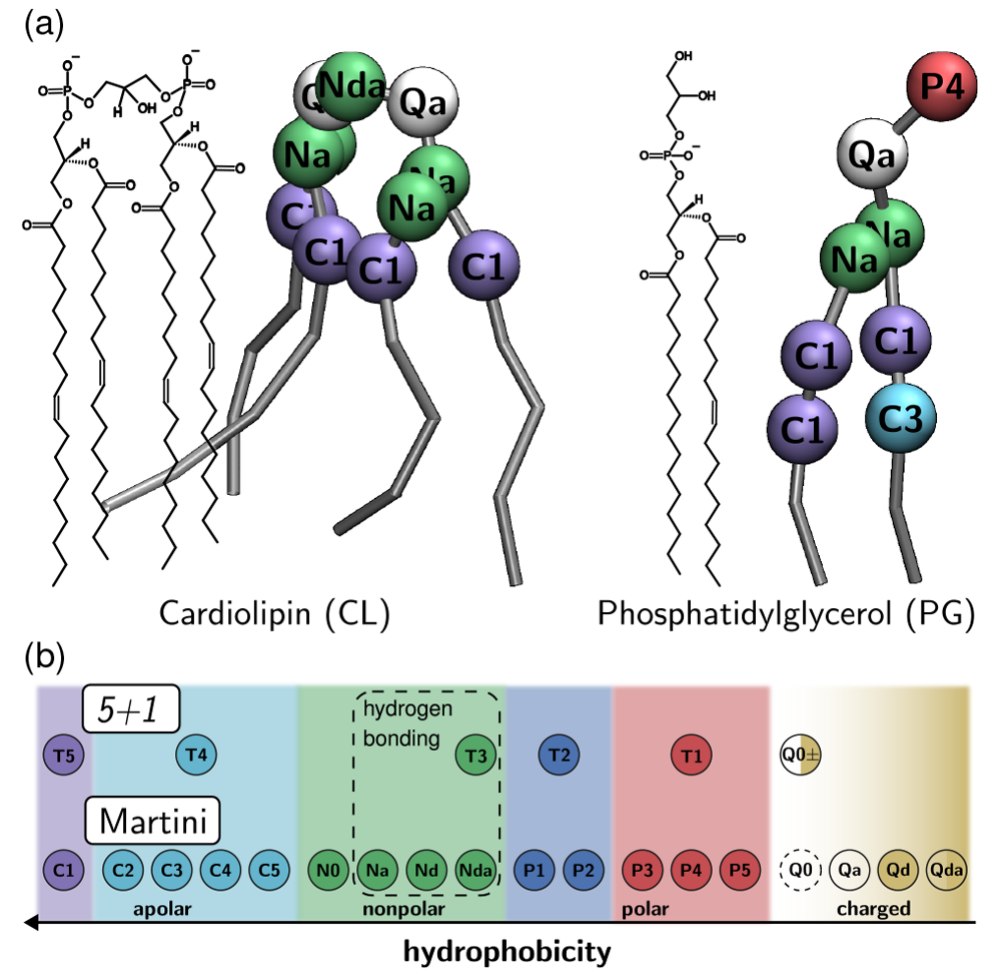
(a) Chemical structures of cardiolipin (CL) and phosphatidylglycerol (PG) next to their coarse-grained (CG) Martini representation. CG beads relevant for solute–lipid interactions are placed according to the chemical structure. (b) CG bead types for Martini and the reduced 5 + 1 force field. The hydrophobicity scale summarizes the beads’ physicochemical characteristics. Structures and cartoon representations were rendered using ChemSketch and VMD.^27,28^

The application we focus on is a challenging biomolecular system: lipid selectivity of small molecules in mitochondrial membranes. The problem involves the subtle identification of preferential interactions between two similar lipids: cardiolipin (CL) and phosphatidylglycerol (PG).^19−26^Figure 2a shows the chemical structures of CL and PG. The binding selectivity of a small molecule between CL and PG membranes amounts to a relative free-energy difference, ΔΔ*G*. Each free-energy difference quantifies the insertion of said compound from bulk water to one membrane interface. Figure 2a highlights the chemical resemblance between one CL molecule and a pair of PG lipids, emphasizing the difficulty of the problem.

The complexity of the system compounded with the size of drug-like chemical-compound space makes an atomistic modeling approach intractable. Instead, we base our investigation on coarse-grained (CG) MD simulations. Coarse-graining averages over atomic degrees of freedom to only describe larger superparticles or beads.^29,30^ Beyond the computational appeal of faster MD simulations, a certain class of CG models has the appealing property of reducing the size of the chemical-compound space.^6,31^ Compressing chemical space translates to a more efficient compound screening, which is a valuable property to establish structure–property relationships. CG models that can reduce the size of chemical space have a top-down parametrization strategy: they aim at modeling large-scale behavior by defining a finite set of bead types, which encode specific physicochemical flavors. Critically, it is the number of bead types that scales the (reduced) size of chemical space of the CG model. While we base our study on the biomolecular CG Martini model,^32,33^ we will use a further reduced yet compatible CG model, made of fewer bead types, to efficiently screen for small molecules.^34^Figure 2b illustrates the reduction in the number of bead types between the original Martini and our reduced so-called 5 + 1 force field. We previously used this approach to devise a rigorous discovery pipeline combining CG simulations, free-energy calculations, and active learning, which, taken together, led to the identification of design rules.^35^ We further showed that such CG simulations can be used to propose small-molecule probes for experimental validation, with exciting results both in vitro and in vivo.^36^

The CG simulations will be used as test system for the ensemble SLATM approach. Our data set consists of *n* = 439 solute small molecules, for which we have calculated the target property, ΔΔ*G*. Here we will run a single isothermal–isobaric MD simulation per compound and lipid environment, so as to compute the averaged structural order parameter. Because the resulting molecular representation is difficult to interpret, we will apply dimensionality reduction on the set of *n* = 439 order parameters. To demonstrate the effectiveness of our representation, we will limit ourselves to a linear method: principal component analysis (PCA). The linearity of the method will also be leveraged through interpretability: we will extract the key two- and three-body interactions that are most relevant to modulate selectivity. The gathered insight will allow us to construct prototypical solutes that optimize for the target property. Finally, we will show that a two-dimensional projection in PCA coordinates displays clearly separated basins of solutes with high and poor CL selectivity, effectively generating a clear structure–property map.

## 2 Methods

In the following, we cover the three methodological parts sketched in Figure 1: (i) molecular dynamics simulations; (ii) structural analysis; and (iii) relating order parameters to thermodynamics.

### 2.1 Molecular Dynamics Simulations

Coarse-grained (CG) molecular dynamics (MD) simulations were run using GROMACS 2020 and Martini parameters tailored to GPU acceleration.^37,38^ We used an integration time step δ*t* = 0.02τ, where τ is the natural unit of time of the model. The simulations were kept at constant temperature (*T* = 300 K) and pressure (*P* = 1 bar) using the Langevin thermostat and Parrinello–Rahman barostat.^39^ Electrostatic interactions were calculated using particle-mesh Ewald summation.^40^

Membranes were generated using the CHARMM-GUI Martini maker.^41^ The cardiolipin (CL) and phosphatidylglycerol (PG) membranes consist of 98 and 118 lipid molecules, respectively. They were solvated in water as well as sodium ions to maintain charge neutrality. Bulk water systems consisted of 974 water beads, as well as sodium and chloride ions to mimick the ion concentration of the membrane systems. More details about the MD simulation setups and parameters can be found in Mohr et al.^35^

#### 2.1.1 Coarse-Grained Modeling

All lipids, water, and ion particles were represented using the standard CG Martini 2 force field with refined polarizable models for water and ions.^32,42,43^ For the solute compounds, we used a reduced and compatible Martini-like CG force field.^34^ As compared to Martini’s 14 bead types, the reduced force field only defines 6 types: 5 neutral and one charged, denoted {T1, T2, T3, T4, T5} and {Q0±}, respectively (i.e., we define a single charged bead type, although the charge can take a positive or negative sign). We herein refer to the reduced model as the 5 + 1 force field. Figure 2b highlights the placement of the fewer CG beads on the hydrophobicity axis. Utilizing fewer bead types compresses the size of chemical space, used here to more efficiently screen across solutes.

Solute compounds were constructed by considering various graph representations and a variety of CG bead types from the 5 + 1 force field.^35^ We limited the number of beads to up to five, to roughly stay within the molecular weight prescribed in Lipinsky’s rule of five for drug-likeness of small molecules.^44^ We applied angles and constraints to the compound structures according to their geometry (Figure S2). This small change in conditions as compared to the previously performed free-energy calculations is warranted due to the dependence of the structural order parameter on nonconflicting particle coordinates. See the Supporting Information for more details on the 5 + 1 force field and solute graph representations.

The subsequent structural analysis of a solute in a membrane environment will monitor CG beads from both force fields:

- All beads from the reduced 5 + 1 force field, so as to screen across the solute’s chemical space, that is, {T1, T2, T3, T4, T5, Q0±}.
- Only some beads from Martini: those involved in describing the CL and PG membrane environments, as well as the water and ion models, that is, {Nda, P4, Qa, Na, C1, C3, POL, PQd}.

The combination yields a set of *N* = 14 different bead types, which will impact the dimensionality of the structural order-parameter vectors described below.

#### 2.1.2 Alchemical Free-Energy Calculations of Selectivity

Our target thermodynamic property is the selectivity of a solute to preferably insert in a CL membrane as compared to a similar PG membrane. Selectivity thus corresponds to a relative thermodynamic affinity between the two membrane environments. We quantify the individual insertions by means of transfer free energies from bulk water to the interfacial region of the membrane bilayer, denoted
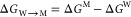
1

Accordingly, selectivity is measured by the difference of transfer free energies between the PG and CL environments:
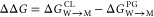
2

Both terms in eq 1 were calculated using relative alchemical free-energy calculations: we focused on the change in free energy when solvating the solute. The water environment was simulated using a simple water box. The membrane simulation consisted of an equilibrated lipid bilayer with added solute placed at the interface, that is, close to the lipid headgroups, which embodies the main chemical difference between PG and CL.

Alchemical free-energy calculations consisted of successive coupling of all nonbonded interactions (i.e., van der Waals and electrostatics) between a solute and its surrounding environment, together with the use of soft-core potentials.^45−47^ We applied 40 intermediate coupling steps for each interaction type to ensure adequate sampling. We subsequently estimated free energies using the MBAR method and the pymbar package.^48,49^ For more details about the free-energy calculations, see Mohr et al.^35^

#### 2.1.3 Trajectory Analysis

The present structural analysis solely relies on the fully coupled alchemical state of the system, while other states were entirely discarded. For each one of the *N* = 439 compounds, we ran and analyzed an MD simulation of total simulation time Δ*t* = 20, 000 τ, and extracted 200 frames. For each snapshot, we centered the simulation around the solute and kept information up to a radial distance of 1.1 nm. Trajectory processing was performed using MDAnlysis.^50,51^

### 2.2 Structural Analysis

#### 2.2.1 The Spectrum of London Axilrod–Teller–Muto (SLATM) Representation: Atomic Case

**Figure 3:**
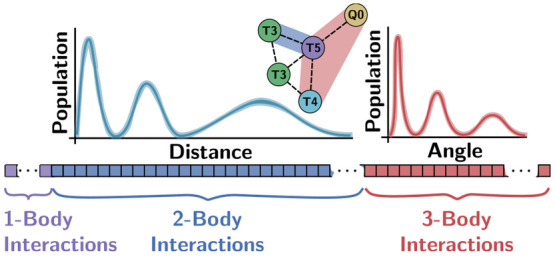
Schematic of a SLATM histogram with decomposition in one-, two-, and three-body contributions: particle counts (purple), pairwise interactions (blue), and triplets (red). Inset: Cartoon representation of interacting beads with example two- and three-body interactions around the T5 particle. The dashed lines emphasize that interactions need not be covariant.

The Spectrum of London Axilrod–Teller–Muto (SLATM) representation describes an atomic environment as a vector of one-, two-, and three-body interactions occurring within a cutoff (Figure 3).^14,15^ SLATM ignores the notion of covalent bonding. The representation features translational, rotational, and permutation invariance. Given a particle *i* (atom or CG bead), let *I* refer to its atom or bead type, one out of *N* types defined by the force field. We denote by ***x***_*i*_ the SLATM representation of particle *i*, as a sum over body-order contributions:

(1) The one-body term, *x*_*i*_^(1)^, simply accounts for the identity of the particle, denoted *Z*_*I*_, the elemental atomic number for particle *i* in an atomistic representation. For the present CG resolution, we remedy the lack of elemental number by assigning *Z*_*I*_ an arbitrary (but unique) value.

(2) The two-body interaction, *x*_*i*,*J*_^(2)^(*r*), represents the population of pairwise interactions between *i* and all other particles of type *J*, as a function of radial distance, *r*.

(3) The three-body bond-angle interaction, *x*_*i*,*JK*_^(3)^(θ), describes the interactions between *i* and all other particles of types *J* and *K*, as a function of the angle, θ, and averaged across interparticle distances.

The radial and angular dependencies of the two- and three-body interactions are binned along their respective intervals: [0, *r*_cutoff_] and . For ease of notation, we represent the binned interaction as a vector. For instance, the two-body representation between particle *i* and all others of type *J* yields
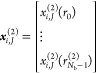
3where *r* denotes the interparticle distance, and the size of the vector is given by the number of radial histogram bins, *N*_b_^(2)^. A similar representation is considered for three-body interactions between particle *i* together with all combinations of types *J* with *K*, ***x***_*i*,*JK*_^(3)^, which would bin over the angle between a triplet of particles. SLATM then concatenates over all possible pairwise and triplet types to yield
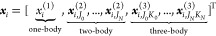
4

Functional forms for two- and three-body interactions follow the London dispersion forces and the Axilrod–Teller–Muto potential.^52−54^ The body-order interactions read
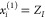
5
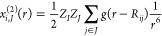
6
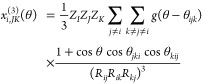
7where *R*_*ij*_ and θ_*ijk*_ are the pairwise distance and triplet angle, respectively, and two- and three-body interactions are smoothened by a Gaussian function:
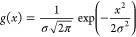
8

We used the implementation of Christensen et al. and adapted some of their parameters for use with CG resolution.^55^ Notably, the widths of the Gaussian kernels were set to σ = 0.3 Å and 0.2 rad for distances and angles, respectively. The bin widths of the histograms were set to 0.2 Å and 0.2 rad, and the radial cutoff was set to *r*_cutoff_ = 8.0 Å.

#### 2.2.2 Boltzmann-Ensemble Averaging

A single configuration is not statistically significant; that is, we require a Boltzmann average of the representation, ⟨***x***_*i*_⟩. By ergodicity, we approximate the Boltzmann-ensemble average by a time average, that is, over snapshots of the MD trajectory. Although we gather 500 equidistant frames along the trajectory, we only keep a subset of 200 to exclude those whose solute lies further away from the target depth of insertion (i.e., at the lipid headgroup interface). We calculate the Boltzmann averages over these 200 snapshots of each atomic SLATM.

#### 2.2.3 Molecular SLATM

Rather than focusing on atomic representations, we describe the behavior of an entire molecule at once. To do so, we sum over all CG beads of a molecule of interest, , so as to yield the Boltzmann-averaged molecular representation:
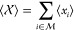
9

The sum in eq 9 requires a separation of contributions in the various bead types. A single atomic SLATM contains 1, *N*, and *N*(*N* + 1)/2 one-, two-, and three-body terms. When summing over multiple particles, the molecular SLATM will feature *N*, *N*(*N* + 1)/2, and a subset of *N*^3^ types, pairs, and triplets, respectively. For the number of triplets considered, see Supporting Information section 2. In this study, we consider *N* = 14 bead types, leading to 14, 105, and 1361 contributions.

Each two- and three-body contribution has high dimensionality, as it is a histogram over a range of distances or angles. To reduce the dimensionality of the molecular SLATM vector, we averaged over the distance and angular information. Averages were normalized against the sum over the corresponding distances or angles (eq S1).

### 2.3 Relating Order Parameters to Thermodynamics

Recall that our target thermodynamic property is a solute’s selectivity to CL and against PG membranes. The following describes our tailoring of the molecular SLATM representation to focus on selectivity and the use of principal component analysis (PCA) to establish a structure–property relation.

#### 2.3.1 Tailoring SLATM to Membrane Selectivity

The power-law behavior used for the two- and three-body interactions leads to strong heterogeneities in the SLATM bins. Order-of-magnitude differences are commonly observed, making their immediate use for any ML analysis potentially difficult. Instead here we work with the logarithm of the molecular SLATM, so as to compress the space.

Focusing on the difference in observed interactions between CL and PG environments, our quantity of interest is the difference between the two log-transformed molecular representations, leading to
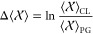
10

For each one of the *n* = 439 herein considered compounds, we computed the structural order-parameter vector, . Further details are included in the Supporting Information.

#### 2.3.2 Principal Component Analysis (PCA)

Each structural order-parameter vector, , is of high dimensionality: *D* = 1480. To reduce the dimensionality and effectively tease out the contributions most relevant to thermodynamic selectivity, we apply a simple methodology: principal component analysis (PCA).^56−59^ PCA looks for a set of orthogonal directions that maximizes the variance of the zero-mean data matrix, ***X̂***, of dimension *n* × *D*, by solving the eigenproblem:
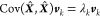
11where λ_*k*_ and ***v***_*k*_ are the *k*th eigenvalue and unit-norm eigenvector, respectively. Similarly, the linear combination ***X̂v***_*k*_ is called the *k*th principal component (PC), a scaled eigenvector. The elements of the eigenvectors ***v***_*k*_ are called the PC loadings.^60^ Intuitively, eigenvectors indicate the directions of high variance in a set of samples, while eigenvalues represent the corresponding amount, via the variance of the PCs. The proportion of variance explained up to dimension *d* is given by *∑*_*i*__<*d*_λ_*i*_/*∑*_*j*__<*D*_λ_*j*_.^61^

The PCA representation then consists of choosing a number of components *d* (where, typically, *d* ≪ *D*), and projecting the original data onto the eigenvectors as ***Y*** = ***XV***, where ***V*** is a matrix of dimension *D* × *d* containing the first *d* eigenvectors. Correlating lower-dimensional PCs to target properties offers strong interpretability, thanks to the possibility to transform back from PCs to original coordinates.^2,61^

We used the PCA implementation of the scikit-learn package with the random seed set to a constant value for reproducibility.^62−64^ We performed no whitening of the data. For computational efficiency, we used the PCA module using randomized singular value decomposition, utilizing the appropriate dimensionality and shape of the SLATM arrays.

#### 2.3.3 PCA of Molecular SLATM Vectors Depends Almost Exclusively on Two- and Three-Body Interactions

Although in principle all three bodies of interaction play a role in the PCA analysis of  (eq 10), the one-body contributions are virtually negligible. Indeed, the two lipid environments display almost the same collections of bead types. The headgroup beads, Nda and P4, are the distinguishing characteristics between CL and PG, respectively (see Figure 2). This difference is systematically present in all . Consequently, PCA places minimal importance on the one-body contributions relative to the higher-order interactions. In the following, we thus limit our evaluation to the two- and three-body interactions.

#### 2.3.4 Physicochemical Interpretation of the Principal Components

Interpretation of the main PCs was achieved by cross-correlation with several (physicochemical) descriptors. All descriptors are normalized by the number of CG beads in the solute, to account for the heterogeneity in solute sizes. The descriptors include the water–octanol partitioning of the solutes, Δ*G*_W→Ol_ (see Supporting Information section 1.1); number of solute polar beads, that is, T1 and T2; number of solute charged beads, that is, Q0; number of solute beads that offer hydrogen-bond-like characteristics, that is, T3; and the *l*^2^ norm of the structural order-parameter vector, . We relied on linear regression to measure the correlation, quantified by the coefficient of determination, *R*^2^.^65^

## 3 Results and Discussion

The following describes the results of the methodology sketched in Figure 1 in the context of small solute molecules interacting with either cardiolipin (CL) or phosphatidylglycerol (PG) membranes. We run MD simulations, extract structural order-parameter vectors (here in the form of the molecular SLATM), and subsequently analyze them using principal component analysis (PCA). We first relate some of the first principal components (PCs) to physicochemical properties. We then focus on the PC most relevant for selectivity and identify key two- and three-body interactions. Finally, we establish linear structure–property relationships between PCs and selectivity for CL membranes.

### 3.1 Physicochemical Interpretation of PCA Eigenvectors

The amount of variance explained by the eigenvalues ideally prescribes a number of PCs to retain *d* ≪ *D*. Upon inspection, we find no clear change in regime, but rather a smooth behavior (Figure S5). We focus here on the first six eigenvectors, representing 77% of the overall variance.

**Figure 4:**
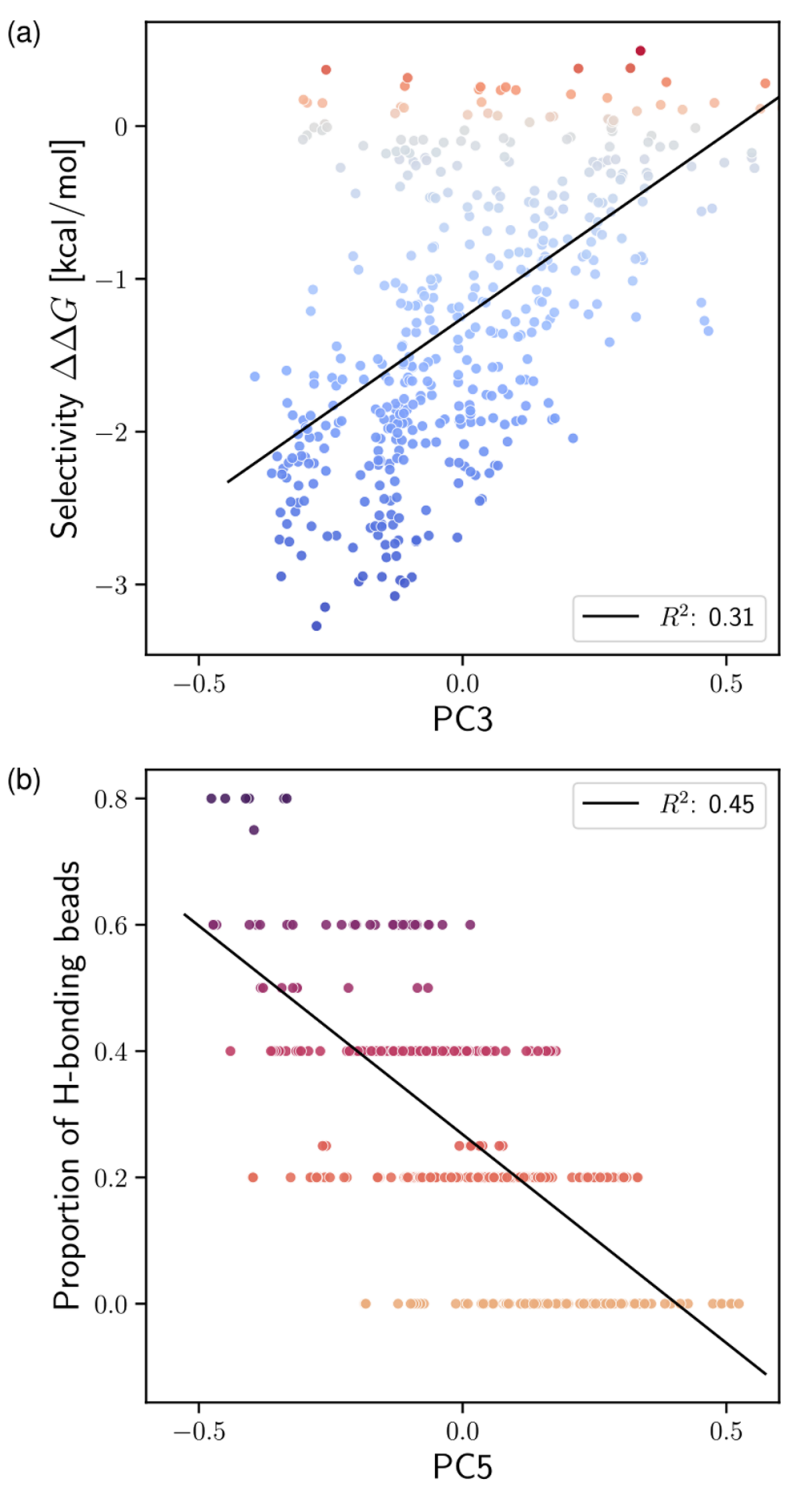
Cross-correlation of (a) the third principal component (PC3) to CL selectivity ΔΔ*G* and (b) PC5 to the ratio of hydrogen-bonding beads in each solute. The color gradients further visualize the respective physicochemical descriptor represented on the vertical axis. The lines represent best fits from linear regression.

To interpret the first six PCs, we cross-correlate them with different physicochemical descriptors. Figure 4a shows the correlation between the third component, PC3, against selectivity itself, ΔΔ*G*. We measure a meaningful coefficient of determination *R*^2^ = 0.31, while cross-correlation with the other main PCs yields virtually 0 (Figure S6). It is not surprising to find correlation between the PCs and the target property, because of our construction of the structural order-parameter vector. Indeed,  focuses on the difference in observed interactions of a solute between the two lipid environments. Although expected, the lack of correlation with any other main PCs makes for a clear map between solute and target selectivity, via a single PC.

Simultaneously, we find that PC3 also correlates strongly with other physicochemical descriptors: water–octanol partitioning free energy, Δ*G*_W→Ol_ (*R*^2^ = 0.36); polar bead types, T1 and T2 (*R*^2^ = 0.57); and charged bead types, Q0 (*R*^2^ = 0.47). However, PC3 does not correlate significantly with bead types T3, associated with a CG proxy for hydrogen bonding (Figures S8–S10). Taken together, the direct association of PC3 to selectivity hints at the role played by Δ*G*_W→Ol_, polar beads, and charged beads in modulating CL selectivity. In fact, Δ*G*_W→Ol_ is a key quantity in the parametrization of CG Martini, and in particular that of the reduced 5 + 1 force field.^34^ The design rules inferred from our previous active-learning study similarly highlighted the effects of polar and charged beads.^35^

Other PCs also exhibit some physicochemical interpretation, as shown in Figures S6–S11. We find that PC1 and PC2 weakly correlate with polar beads (*R*^2^ = 0.14) and hydrogen-bonding beads (*R*^2^ = 0.13), respectively. The other main PCs correlate more significantly to physicochemical descriptors: PC4 associates with both water–octanol partitioning (*R*^2^ = 0.22) and charged beads (*R*^2^ = 0.11). PC5 strongly correlates with T3 types associated with hydrogen bonding (*R*^2^ = 0.45, Figure 4b), and to a smaller extent with the norm of the structural order-parameter vector (*R*^2^ = 0.32) as well as the number of charged beads (*R*^2^ = 0.21). Finally, PC6 almost exclusively and strongly correlates with the norm of the structural order-parameter vector (*R*^2^ = 0.51). It is not clear to us whether this mirrors a sensitivity to an overall difference between CL and PG environments, or whether the metric is biased by particular coordinates of .

Overall, the relatively straightforward association of PCs to few physicochemical descriptors likely arises from the CG resolution of the model itself, which reduces the number of relevant degrees of freedom. In addition, we point at the effective role played by our MD structural order-parameter vectors, which exacerbates the relationship between salient features of the solute in its condensed-phase environment with the target thermodynamic property.

### 3.2 Identification of Key Interactions to Design Selective Solutes

Correlation of relevant PCs to selectivity is only a means to an end. What we care to understand is the role played by specific (two- and three-body) interactions in modulating selectivity. Fortunately, the linearity of PCA allows us to easily transform back to the space of  and read off the contribution of every single interaction. To this end, we focus on the scaled PC loadings (eq S2), that is, the elements of the PCA eigenvectors. The scaled PC loadings for the various PCs down to an absolute value of 1.0 are reported in Figures S12 and S13. However, not all components carry equal importance. Recall from Figure 4a that PC3 correlated positively with selectivity. However, strong selectivity values tend to be large and negative (i.e., they are free-energy differences). Given the positive correlation between PC3 and ΔΔ*G*, we expect PCs with negative coefficients to contribute to strong selectivity.

**Figure 5:**
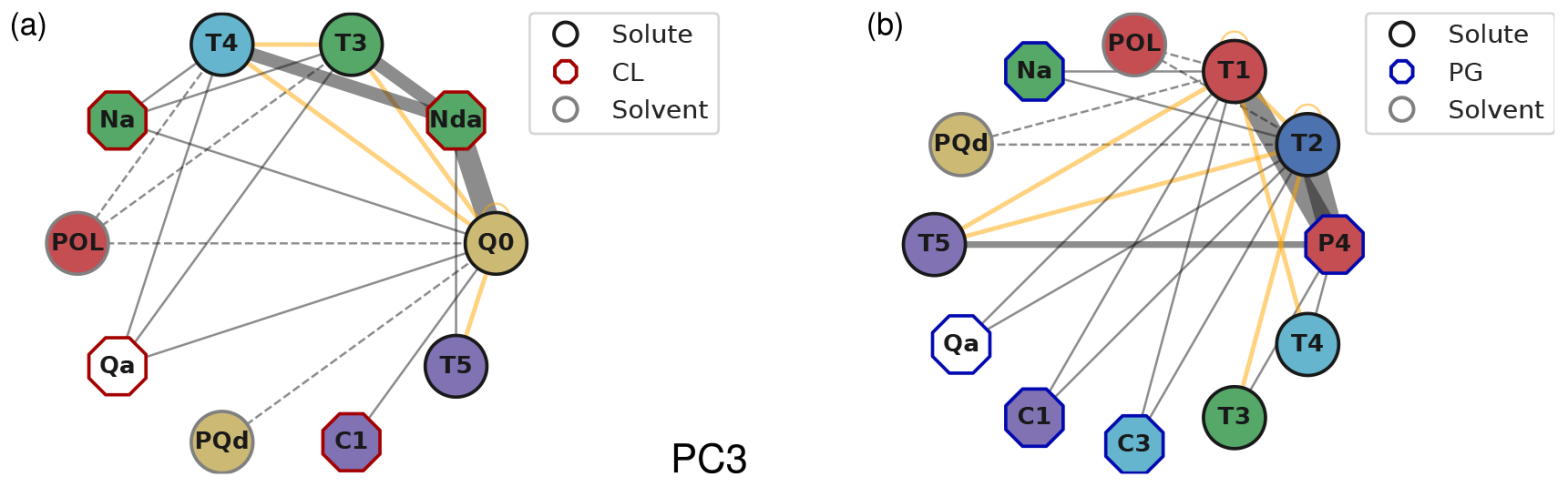
Graph of the interactions of PC3 with dominant negative PC loadings for the (a) CL and (b) PG systems. The edge width scales with the occurrence of the interaction. Orange edges show interactions between solute beads, and dashed edges represent interactions with beads used to model water or sodium ions.

Figure 5 reports the interactions that have negative PC3 eigenvector components (see the Supporting Information for the other PCs). The interactions are displayed on a graph, where nodes and edges correspond to bead types and interactions, respectively. Because edges are inherently pairwise, three-body interactions are projected down onto the relevant pairwise counterparts. Both bead types and interactions have specific visual features depending on the system: solute, lipid, or solvent. Importantly, the thickness of the edges emphasizes the occurrence of a bead pair in the dominant scaled PC loadings, and thus the relevance of the interaction. Panels a and b display the CL and PG systems, respectively. First, they highlight the central role played by the Nda and P4 lipid beads. We recall from Figure 2a that these are the two bead types that specifically distinguish CL from (2×) PG. For CL, Nda predominantly interacts with Q0, T3, and T4. For PG, P4 interacts primarily with T1 and T2. For both membranes, these contributions largely reflect the strengths of two-body interactions. In addition, they are further reflected in many of the relevant three-body interactions, sometimes accompanied by other bead types: T5 for CL; T3, T4, and T5 for PG. Furthermore, the role of the solvent is highlighted via key interactions with the POL and PQd bead types.

**Figure 6:**
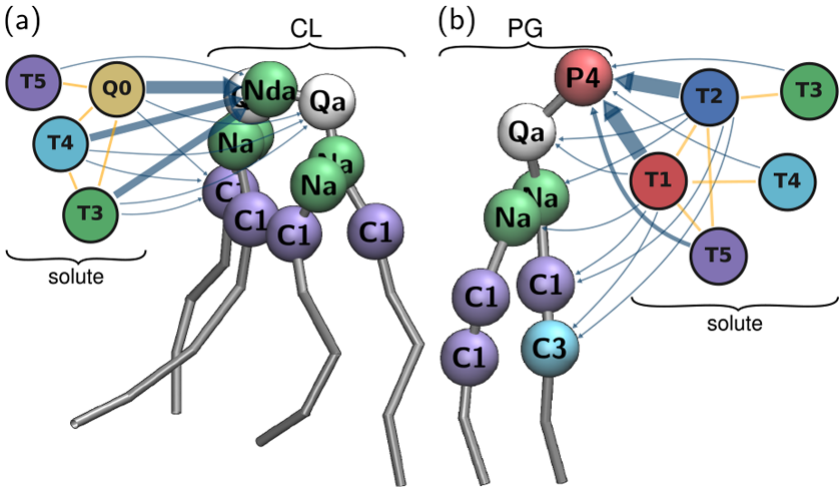
Three-dimensional illustration of the dominant interactions of PC3 reported in Figure 5 for (a) CL and (b) PG. The sets of beads form hypothetical solutes that would favorably interact with either lipid.

Leveraging information from this analysis further, we can visualize favorable geometric arrangements of beads to enhance selectivity. Figure 6 reconstructs information from the interaction graphs to place prototypical solutes around the two lipids. Solute beads are placed manually around the lipids so as to illustrate the information of Figure 5. The arrow widths further reflect the interaction strengths, mirroring the PC loadings. The figure emphasizes the role played by some of the bead types and clearly conveys the idea that different bead types will favorably associate with either CL or PG. Panel a, which targets CL, better illustrates relevant solute characteristics for the target property at hand in this work.

### 3.3 Charting Selectivity in Low-Dimensional Maps

**Figure 7:**
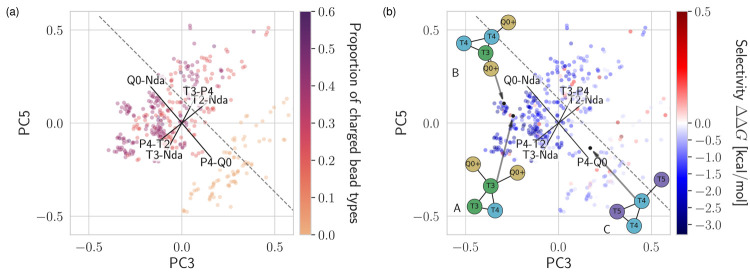
Biplots of PC3 and PC5, colored by (a) the ratio of charged beads per solute and (b) the selectivity, ΔΔ*G*. The values of the principal components are scaled to the interval [−1, 1]. In (a) and (b), we show the six highest eigenvector coefficients of two-body interactions. In (b), three example compounds are classified for CL selectivity by PCA, without calculating their respective partitioning free-energy difference ΔΔ*G*.

Beyond the relationship between individual PCs and selectivity, we look for more insight by combining pairs of components. We iterate through all pairs of PC1–6, each time generating a two-dimensional map or embedding, populating it with the *n* = 439 solutes based on their PCA coordinates, and coloring the points according to the different physicochemical descriptors (Figures S21–S32). Out of all combinations, the pair PC3–PC5 stands out in its high overall correlation to several descriptors, including selectivity. The two-dimensional map is reproduced in Figure 7a. The combination is somewhat expected: the high correlation of PC3 and PC5 alone was already reported in Figure 4a and b, respectively. Figure 7a shows that the combination of PC3–PC5 creates two clear basins in terms of proportion of charge in the solute. Remarkably, this projection simultaneously leads to a separation between poorly and highly selective solute compounds, as evidenced in Figure 7b. This separation is clearly visible between the upper-left and lower-right corners of the space. The basin of high selectivity associates with low and high values of PC3 and PC5, respectively.

The two-dimensional maps of Figure 7 represent so-called biplots, because they also feature the directions and magnitudes of the PC loadings via the displayed arrows. Intuitively, each arrow points at the correlation between a select two- or three-body interaction and a PC. Figure 7 focuses on two-body interactions only and clearly highlights how the P4–Q0 and Q0–Nda both align perpendicular to the separation between poor and high solute selectivity. The biplot further singles out the role of Q0 as a key solute bead to modulate selectivity, but specifically identifies the bead’s impact in terms of two-body interactions.

Now that we have a two-dimensional map charted with clear basins of poor and high selectivity, we apply it to predict the selectivity of new solutes. We construct three compounds outside of the initial set of *n* = 439. Compounds A and B follow the three-dimensional structural aspects prescribed by Figures 5a and 6a; that is, they are expected to be selective to CL. Compound C, however, was originally eliminated from our initial study because of a lack of stable insertion at the membrane interface, that is, expected to not be selective to CL.^35^ For these three compounds, we have no free-energy calculation to determine ΔΔ*G*. We will instead solely apply the methodology sketched in Figure 1: run a single MD simulation, compute the structural order-parameter vector across the trajectory, transform  to PCA coordinates, and place the new compound on the two-dimensional PC3–PC5 map.

Figure 7b places the three compounds A, B, and C on the two-dimensional map. C is featured well within the basin of poorly selective compounds, where its lack of charged beads places it toward the lower-right side of the map. Compounds A and B, however, stand to the upper left of the dividing line between poor and high selectivity, suggesting selectivity to CL. For both compounds, the presence of charged beads places them toward the leftmost side of PC3, while the number of T3 beads likely impact the different positions along PC5. Naturally, a larger set of compounds would enrich the chemical space explored, but even within our limits, we achieve reasonably accurate predictions.

Evidently, estimating selectivity by transforming the solute’s  to PCA coordinates offers significant appeal in terms of computational load. The alchemical free-energy calculations involved in calculating ΔΔ*G* consumed from 24 to 48 GPU hours of an NVIDIA Tesla V100 per neural and charged compounds, respectively.^35^ However, a single MD simulation used in the present protocol only needed 0.3–0.7 GPU hours of an NVIDIA GTX 980. Although the two GPUs are different, the need for a sole MD simulation, and without the usual sensitivities associated with alchemical free-energy calculations, evidently leads to a drastic reduction in computational load.

## 4 Conclusion

The present work proposes a methodology based on molecular simulations to link chemical structure to thermodynamic properties. Attempting a direct structure–property link, for example, via machine learning, between chemical compound and target property is likely to be clouded by several factors. First, the condensed-phase environment of a liquid will likely lead to a combination of covalent and noncovalent interactions, and both may critically impact the target property. In addition, a single three-dimensional molecular configuration is unlikely to be representative, because of the phase-space (Boltzmann) averaging inherent to thermodynamic quantities.

To address this challenge, we propose the use of an atomic representation originally developed for machine learning of electronic properties: the Spectrum of London Axilrod–Teller–Muto (SLATM) representation. SLATM decomposes a configuration into a collection of increasing body-order interactions: single particle (one-body); pairwise (two-body); and triplets (three-body). For each term, SLATM builds a histogram of population of these interactions. The pairwise term is reminiscent of the radial distribution function, which hints at the adequacy of the representation for molecular liquids. We adapt SLATM to average over snapshots of an isothermal–isobaric MD simulation, acting as a proxy for a Boltzmann average. This adapted ensemble-SLATM representation thereby addresses the two above-mentioned issues: (i) it does not distinguish between covalent and noncovalent interactions; and (ii) it offers phase-space averaging.

We argue that this adapted ensemble-SLATM representation is particularly amenable to establishing structure–property relationships of thermodynamic properties. As an application, we focus on a complex biomolecular system: small molecules targeting (phospholipid) cardiolipin (CL) membrane environments. We rely on a coarse-grained (CG) resolution, not only for computational efficiency, but mostly for its ability to reduce the size of chemical space and thereby screen across compounds more efficiently. The CG resolution allows us to screen across a large subset of small drug-like molecules with relatively few CG molecular structures. Although based on the biomolecular CG Martini model, our solute compounds are represented via a further reduced force field that defines fewer bead types.

Establishing here the structure–property map boils down to reducing the dimensionality of the SLATM vectors. To demonstrate the benefits of including relevant physics in the representation (e.g., phase-space averaging or key two- and three-body interactions), we apply a simple, linear statistical method: principal component analysis (PCA). Transformation of the original coordinates to the main principal components allows us to focus on a handful of dimensions, thereby significantly reducing the dimensionality of the problem.

Our analysis shows that we can correlate the first main principal components (PCs) against relevant physicochemical descriptors, as well as CL selectivity, the target property itself, via a single PC. The linearity of PCA makes it possible to transform back from PCA to SLATM coordinates to identify key two- and three-body interactions that impact the various PCs. We isolate key CG bead types present in higher-order interactions that overwhelmingly impact CL selectivity. In the present case, this includes CG types Q0, T3, T4, and T5, interacting favorably with the Nda bead type on CL’s headgroup. The results offer direct prescriptions on the design of solutes selective to CL.

Finally, we gain further insight by charting a two-dimensional map in the PCA coordinates. A simple evaluation of all pairs of PCs reveals one that surprisingly separates two clear basins of compounds: poor and high CL selectivity. From this map, it is straightforward to predict a compound’s thermodynamic CL selectivity based on its PCA coordinates. Computationally, this methodology only requires a (relatively short) MD simulation, as compared to expensive alchemical free-energy calculations. We demonstrate the idea on three test compounds out of the initial training set.

Although demonstrated on a CG model applied to CL-membrane selectivity, we foresee the methodology to be generally applicable to molecular simulations of a variety of thermodynamic properties.

## Supporting Information Available

Coarse-grained force field; additional technical information on the preparation of the samples; full set of plots and illustrations generated during the analysis and interpretation of PCA (PDF)

## Notes

MD-trajectories and simulation parameter files; SLATM representations; codes for generating the SLATM representations, performing PCA and analysis of the results can be found on Zenodo: https://zenodo.org/record/7639223; Codes for generating the SLATM representations, performing PCA and interpretation of the PCA can be found on GitHub: https://github.com/Bernadette-Mohr/STRUCTURAL_ANALYSIS.
