# Supplementary material for: Condensed-Phase Molecular Representation to Link Structure and Thermodynamics in Molecular Dynamics
Source: J Chem Theory Comput. 2023 Jul 3;19(14):4770–9. doi: 10.1021/acs.jctc.3c00201 (PMC10373487; doi:10.1021/acs.jctc.3c00201)
Supplement: Supplementary file 1 — ct3c00201_si_001.pdf [file ct3c00201_si_001.pdf]

Supporting Information:
Condensed-phase molecular representation to link structure and thermodynamics in molecular dynamics

Bernadette Mohr*[1], Diego van der Mast[1], and Tristan Bereau[1, 2, 3]

[1]Van 't Hoff Institute for Molecular Sciences & Informatics Institute, University of Amsterdam
[2]Institute for Theoretical Physics, Heidelberg University, 69120 Heidelberg, Germany
[3]Max Planck Institute for Polymer Research, 55128 Mainz, Germany

June 28, 2023

1 Coarse-Grained Representations

We analyzed a data set of MD trajectories with partitioning free energies of coarse-grained (CG) solutes.[1] To efficiently screen for small molecules, we used a Martini-compatible force field with reduced resolution defining five bead types (*5 + 0*).[2] We extended this reduced representation by a charged bead type (*5 + 1*) to capture the overall physicochemical properties of small molecule space and to automatically generate solute structures without requiring extensive reparameterization for each candidate. The bead types of the Martini 2 and the reduced resolution force field are shown in Figure S1. The solutes were systematically generated in the form of graphs (Figure S2). The majority of

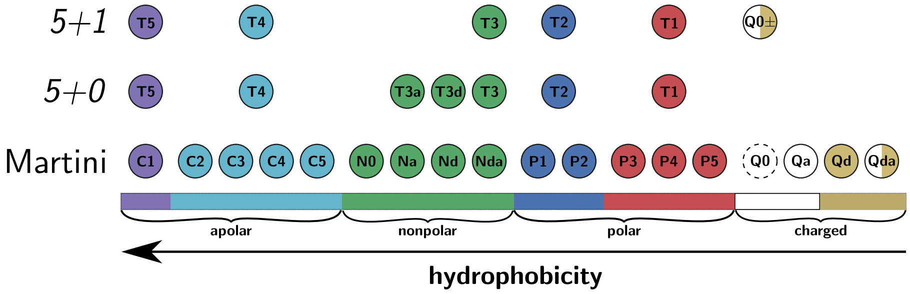

Figure S1: Coarse-grained *5 + 1* model used to represent the candidate solutes. Created by extending the Martini–compatible five-bead-type reduced resolution force field (*5 + 0*) to include a charged bead type Q0± that can carry one positive or negative charge. [1, 2] The bead–types defined in the reduced–resolution force fields are positioned according to their physicochemical properties relative to the Martini bead–types.[3] The T3 bead retained from the *5 + 0* model represents both hydrogen-bond donor and acceptor properties. Figure reproduced from [1] with permission from the Royal Society of Chemistry.

the variation in the data set is based on the permutation of the different bead types along the nodes in a specific graph. We inserted the angles shown in Figure S2 to preserve the solute graph's spacial structure and prevent multiple beads from occupying identical coordinates in the same frame of an MD trajectory. Where applicable, we adopted the settings from Martini solvent and amino acid models. All other decisions were made purely based on geometric considerations. The count number shows the frequency with which each graph structure is present in the data set.

*b.j.mohr@uva.nl

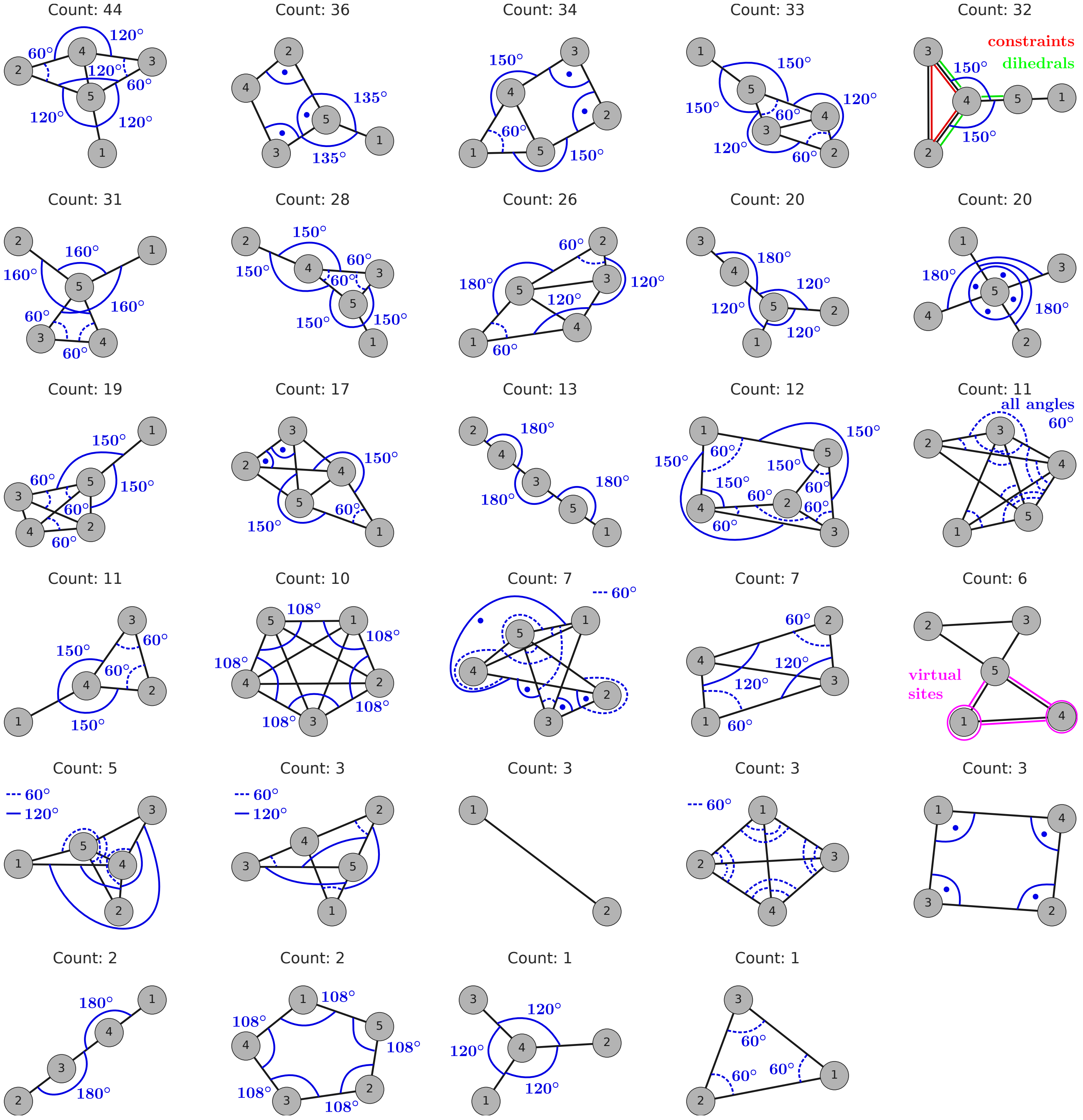

Figure S2: Illustration of the graph structures found in the analyzed samples. The angles were selected to stabilize the compounds and prevent two or more CG beads from occupying identical coordinates within the same frame of a trajectory. Where applicable, examples from the Martini solvent and amino acid models were used. Otherwise, choices were made purely based on geometric considerations. The count number indicates the frequency with which each graph structure is present in the data set.

1.1 Calculation of solute water-octanol partitioning coefficients $\Delta G_{\mathrm{W}\rightarrow\mathrm{Ol}}$

The overall water-octanol partitioning coefficient $\Delta G_{\mathrm{W}\rightarrow\mathrm{Ol}}$ for each solute used for the physicochemical interpretation of the principal components is calculated by summing over the $\Delta G_{\mathrm{W}\rightarrow\mathrm{Ol}}$ parameter of each CG bead type i present in a solute and normalizing the result by the number of beads n forming the entire solute:

$$\Delta G_{\mathrm{W}\rightarrow\mathrm{Ol}} = \frac{1}{n}\sum_{i=1}^{n} \Delta G_{\mathrm{W}\rightarrow\mathrm{Ol},i}$$

The water-octanol partitioning coefficients of each bead type were calculated following Kanekal and Bereau.[2]

**Bead type**	$\Delta G_{\mathrm{W}\rightarrow\mathrm{Ol}}$ [kcal/mol]
T1	2.052
T2	1.906
T3	0.098
T4	-2.455
T5	-3.129
Q0±	20.479

The values are also included in the form of a dictionary `dG_w_ol.pkl` with the GitHub repository accompanying this publication.[4]

2 Determination of admissible three-body interactions by the SLATM representation

Other than in the case of two-body interactions, potential three-body interactions can not be described as a multiset $\binom{N+k-1}{k}$ with length k and number of elements N. For example, two sets $\{a, a, b\}$ and $\{a, b, a\}$ are equal, whereas the sequences of particles (a, a, b) and (a, b, a) describe two different three-body interactions. Therefore, the potential three-body interactions according to the SLATM representation are determined as follows:

1. The total number of possible combinations of length k from a set of N discrete elements, considering both the replacement and ordering of these elements, is given by N^k.

2. The many-body interactions described in the SLATM representation are rotationally invariant. Therefore a pattern x_i is not explicitly included in the results, if the particle sequence of x_i in reverse order is equal to the particle sequence of any of the previously encountered patterns $\{x_1, \ldots, x_{i-1}\}$. A pattern (b, a, a) is considered equal to the pattern (a, a, b) for example.

3. Three-body patterns are excluded from the data set, if any of their constituting particle types is present more often than in any of the compounds that form the analyzed data set. In the exemplary case of a data set containing only compounds with at maximum one oxygen atom, all patterns containing two or more oxygen atoms would be excluded from the set of potential three-body interactions by the implementation of the SLATM representations used in this work. [5]

For our purposes, we have:

1. Combinatorics with replacement: $N^k = 14^3 = 2744$;

2. Remove rotational duplicates: 1470;

3. Correct for the frequency of particle types: 1361.

3 Data Preprocessing

3.1 Weighted arithmetic mean over many-body interaction spectra

To obtain a single value in place of the spectra for each many-body interaction in an ensemble-averaged molecular SLATM representation $\mathcal{X}$, we calculate weighted arithmetic means over the values within the observation range.[6] The individual weights are normalized by the transformation $w_i' = \frac{w_i}{\sum_{j=1}^{n} w_j}$, so that all weights sum up to $\sum_{i=1}^{n} w_i' = 1$. Consequently, the formula for the weighted arithmetic mean simplifies to:

$$\langle \mathcal{X} \rangle = \frac{\sum_{i=1}^{n} w_i x_i}{\sum_{i=1}^{n} w_i} = \frac{\sum_{i=1}^{n} w_i' x_i}{1} = \sum_{i=1}^{n} w_i' x_i. \tag{S1}$$

As the data set used in this analysis contains 14 different CG bead types, a sum of 1480 possible interactions has to be considered. The 1480 interactions comprise 14 1-body interactions, 105 two-body interactions, and 1361 three-body interactions. For admissible interactions not found in a particular system, the corresponding section of the vector is padded with zeros by the SLATM implementation and retains a value of zero in place of a weighted arithmetic mean.

3.2 Normalization of the Data

Principal component analysis (PCA) is highly sensitive to the shape of the analyzed data.[7, 8, 9] Variables with different scales or outliers caused by external processes affect the variance contained in each observed feature. It is therefore of high priority to appropriately scale the data before applying PCA. The logarithmic distribution of the data is clearly visible in the ensemble averages of the SLATM representations. Figure S3 shows the arithmetic means of the bin populations in the SLATM representations for (a) cardiolipin (CL) $\langle \mathcal{X} \rangle_{\mathrm{CL}}$ and (c) phosphatidylglycerol (PG) $\langle \mathcal{X} \rangle_{\mathrm{PG}}$. In order to log-normalize the representations, we first added a minute ε to SLATM bins corresponding to interactions that were not present in either environment (padded with zeros). Figure S3 (b) shows the distribution of the averaged interaction frequencies in the CL environment after log normalization ($\log(\langle \mathcal{X} \rangle_{\mathrm{CL}})$), Figure S3 (d) shows the same for the PG environment ($\log(\langle \mathcal{X} \rangle_{\mathrm{PG}})$). Our objective is to identify the compound structures that show the highest selectivity for interacting with the target CL. We use the structurally very similar PG as a comparison to efficiently identify the minute details CL selectivity depends on. To make this clearly evident in the data we use for our further analysis, we subtracted the SLATM representation representing the many-body interactions in the PG environment from its counterpart representing the CL environment ($\Delta\langle \mathcal{X} \rangle$). As a side-effect of analyzing the distance vector $\Delta\langle \mathcal{X} \rangle$, the ε introduced to facilitate log-normalization is largely removed, minimizing its influence on the results of the downstream analysis. The distribution of the interaction frequency in the $\Delta\langle \mathcal{X} \rangle$ is shown in Figure S4.

3.3 Selection of the Main Principal Components

The principal components (PCs) identified by PCA are sorted by the amount of variance in the transformed, low-dimensional data-set they explain. This can generate a clear picture of their importance. The explained variance of the first ten PCs is shown in Figure S5. We chose to analyze the first six PCs more closely, the following four PCs each contribute little additional variance (left vertical axis). The first six components together explain $\sim 77\%$ of the overall variance in the data, as indicated by the black line in Figure S5 corresponding to the right vertical axis.

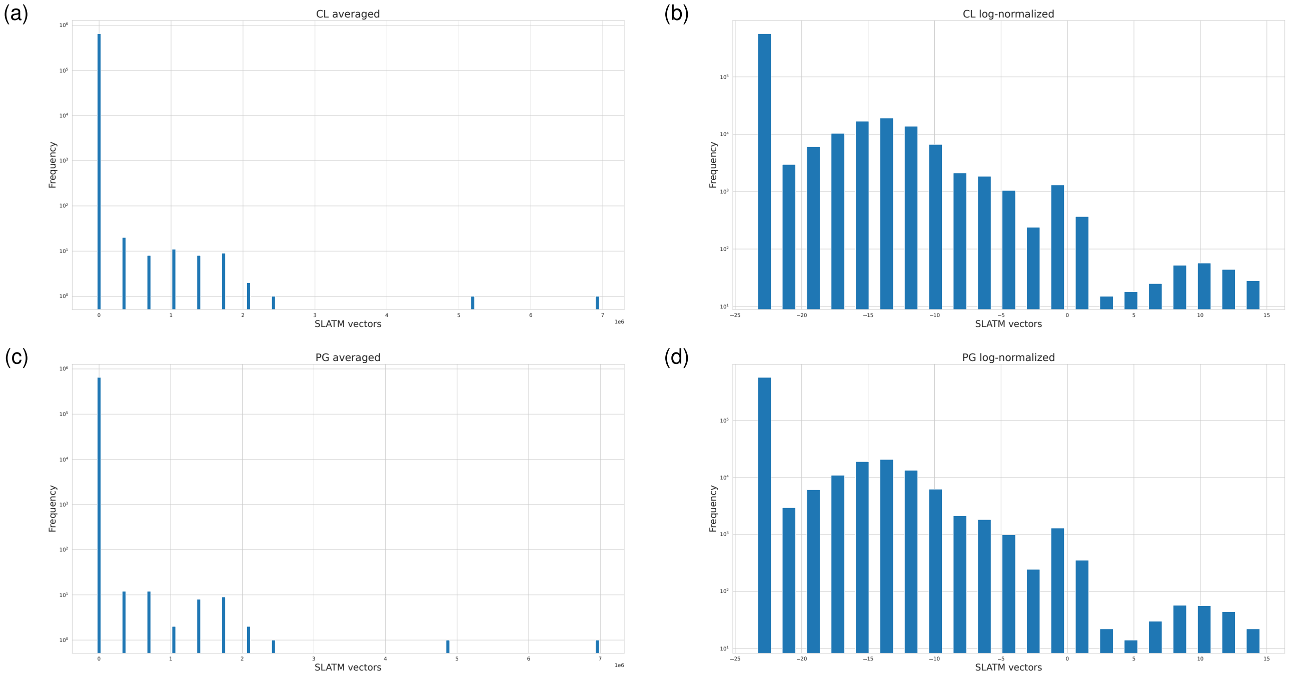

Figure S3: Distribution of the weighted arithmetic means over the interaction frequencies in the SLATM representations. Logarithmic distribution is clearly visible both in (a) the CL environment and (c) the PG environment. The mean interaction frequencies after logarithmic normalization for CL and PG are show in (b) and (d), respectively.

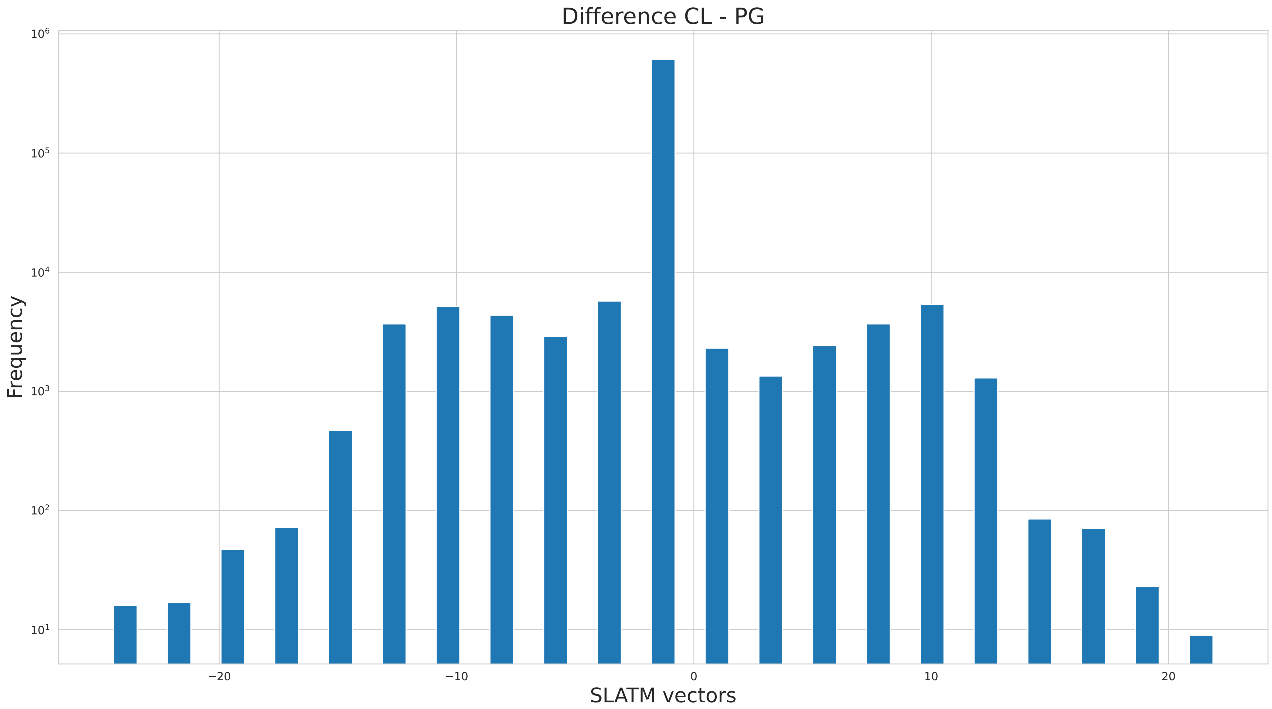

Figure S4: Difference vector $\Delta\langle\mathcal{X}\rangle$ obtained by subtracting the mean log-normalized interaction frequencies of PG from these of CL. The final state of the SLATM representations analyzed by principal component analysis (PCA).

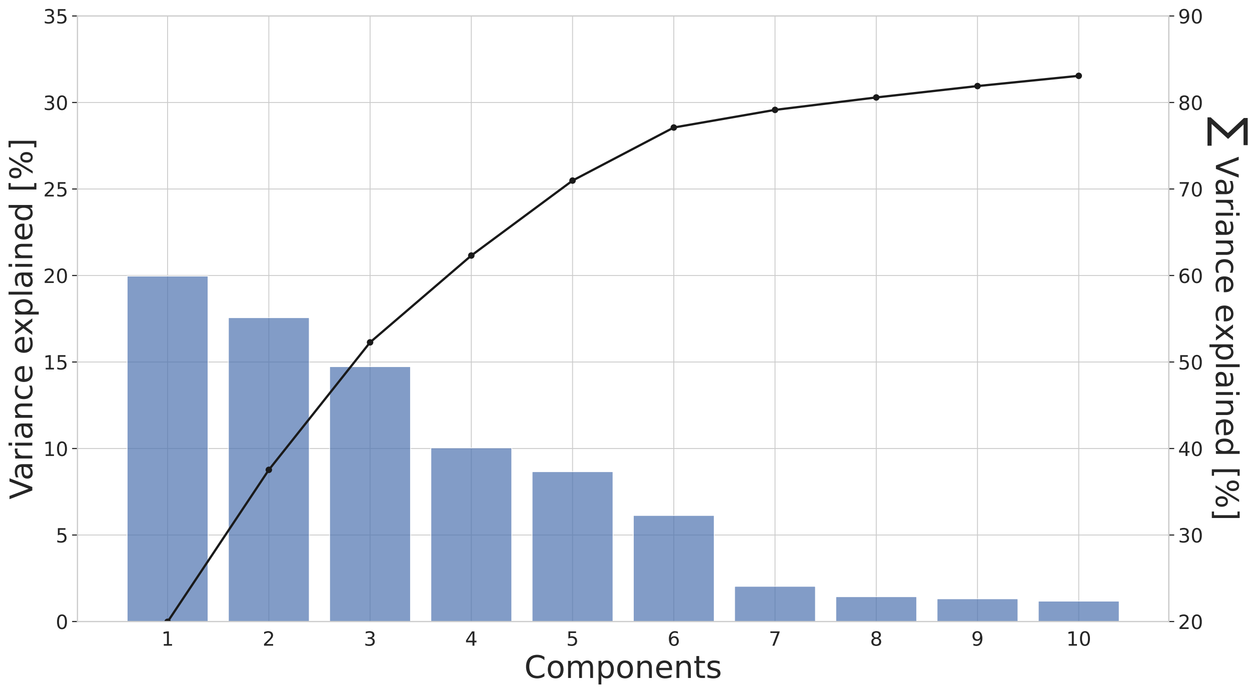

Figure S5: Scree Plot showing the amount of variance in the data set explained by the first 10 components identified by PCA. The principal components are positioned on the horizontal axis, sorted by amount of explained variance. The amount of variance in percent for each component is indicated on the left vertical axis, the cumulative variance explained by the principal components is visualized by the black line corresponding to the right vertical axis.

4 Complete Set of Cross-correlations

We apply cross-correlation to identify the association of individual PCs with different descriptors related to our data set. Linear regression indicates the extend of this association for each case, the coefficient of determination R^2 is provided as a measure of the goodness of fit. Figure S6 shows the cross-correlation between the first six PCs and the partitioning free energy difference $\Delta\Delta G$ we used as a measure for CL selectivity in our previous study.[1]

The strongest correlation is found with PC3. The average hydrophobicity of the candidate solutes, measured by the average water-octanol partitioning coefficient $\Delta G_{\mathrm{W}\rightarrow\mathrm{Ol}}$, is shown in Figure S7. We find that the average $\Delta G_{\mathrm{W}\rightarrow\mathrm{Ol}}$ is again correlated mainly to PC3, weaker correlations can additionally be seen in both PC4 and PC5. The number of polar sites in the solutes (bead types `T1` and `T2`), normalized by the number of beads per solute, is most closely correlated to PC3, with a weaker correlation also visible to PC1 (Figure S8). The average number of charged bead types `Q0` is again correlated to PC3, and to a lesser extend to PC4 and PC5 (Figure S9).

The correlation between the average number of hydrogen bond donor- and acceptor beads (bead type `T3`) and the six main PCs is shown in Figure S10. In this case, we find the strongest correlation to PC5 and a considerably weaker correlation to PC2.

The Euclidean distance (L^2-norm) of the difference vector $\Delta\langle\mathcal{X}\rangle$ quantifies the difference in detected many-body interactions between the CL and the PG environments. The strongest correlation here is found with the lastly evaluated PC6. A weaker correlation can also be observed with PC5.

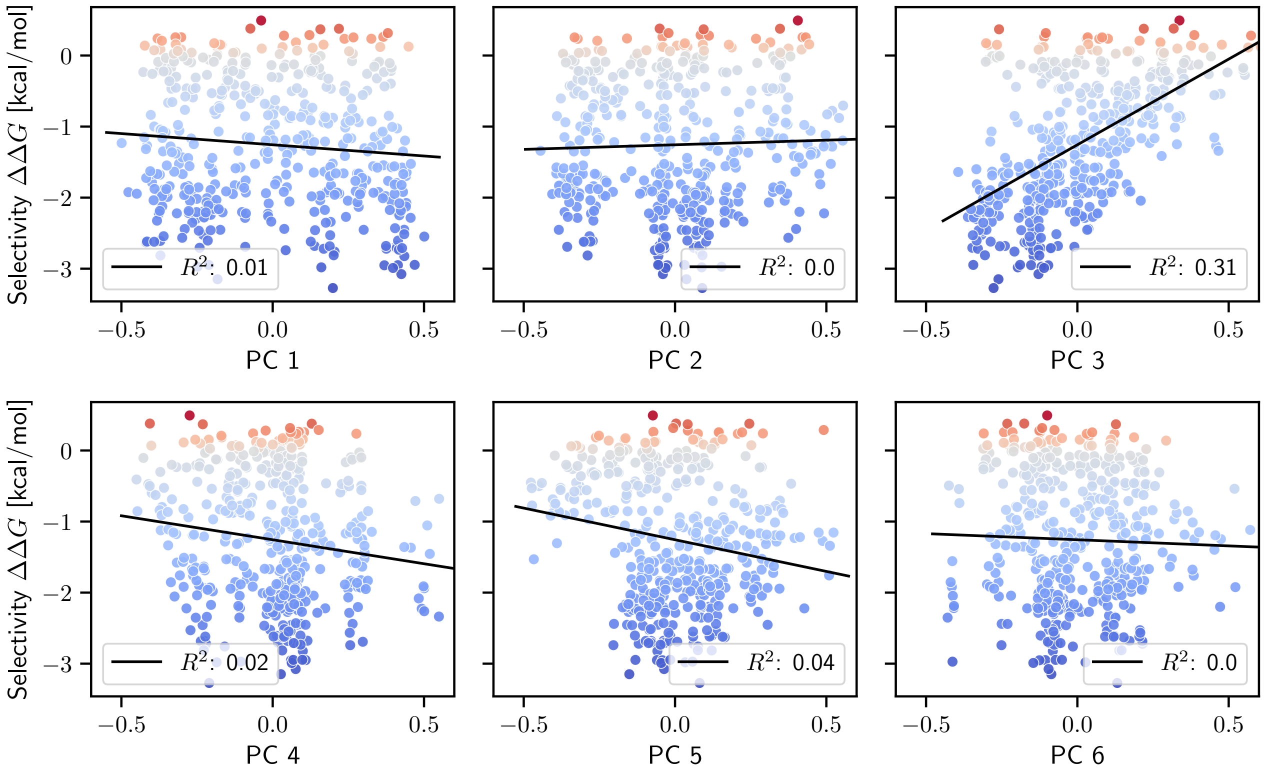

Figure S6: Cross-correlation of the first six principal components and the selectivity metric ($\Delta\Delta G$), the difference in partitioning free energies of a solute into a CL- and a PG membrane, respectively. The strongest correlation is visible with PC3. The color gradient visualizes the selectivity $\Delta\Delta G$.

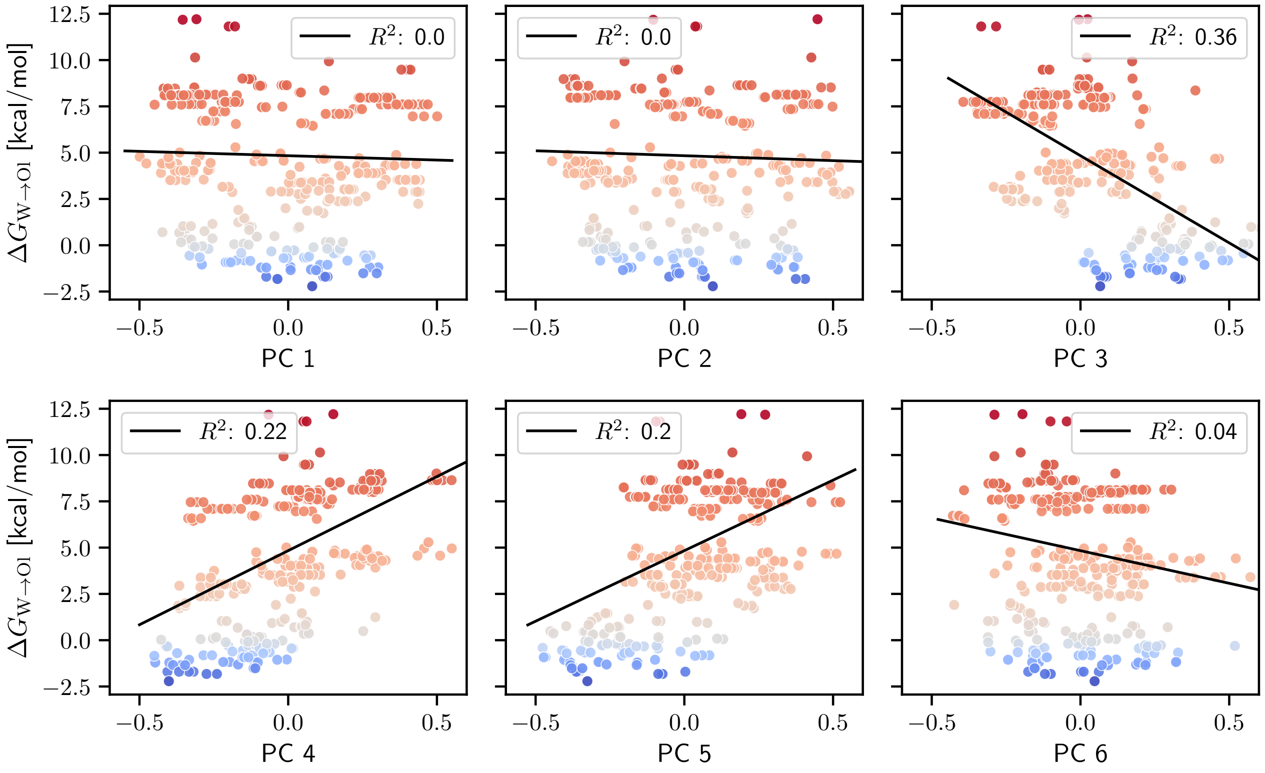

Figure S7: Cross-correlation of the first six principal components and the average water-octanol partitioning free energy of the solutes $\Delta G_{\mathrm{W}\to\mathrm{Ol}}$. We find the strongest correlation with PC3, weaker correlations can also be seen with PC4 and PC5. The color gradient represents the average $\Delta G_{\mathrm{W}\to\mathrm{Ol}}$.

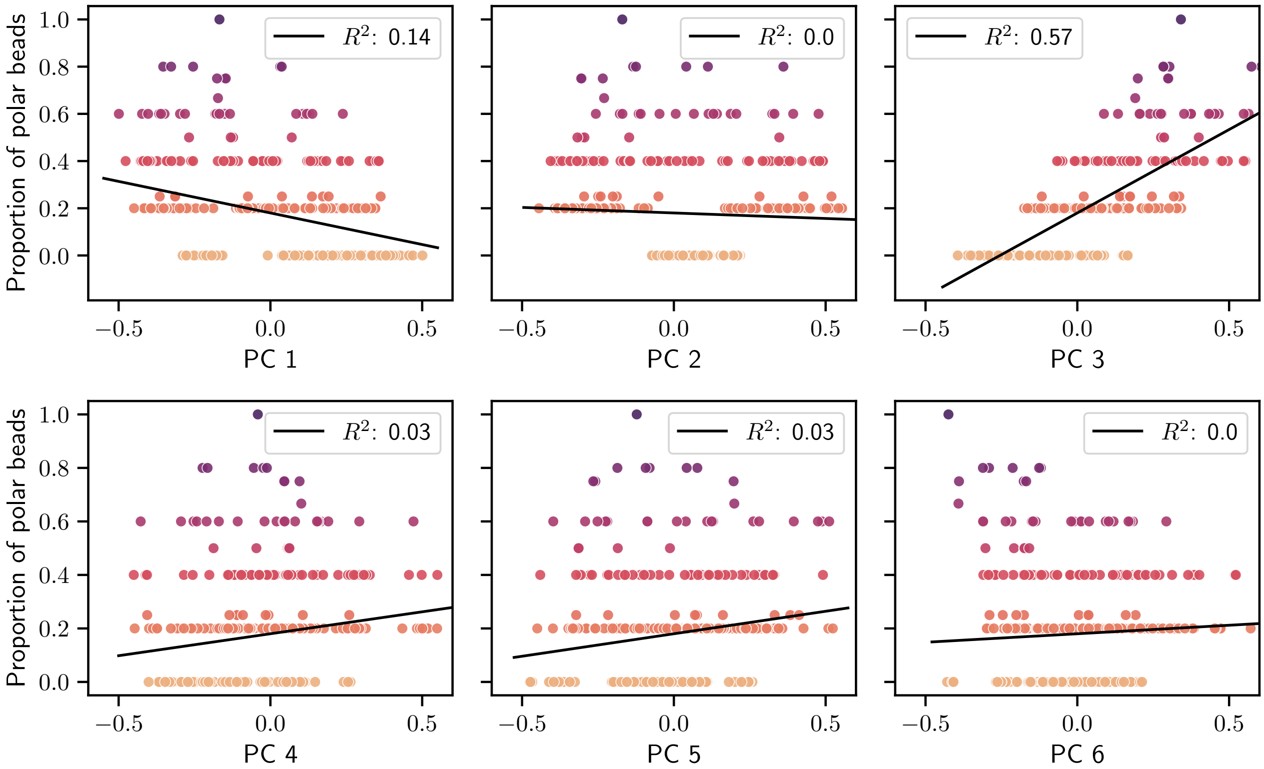

Figure S8: Cross-correlation of the first six principal components and the normalized number of polar beads per solute (T1 and T2). The by far strongest correlation is again found with PC3, a considerably weaker correlation is also visible with PC1. Color gradient: the normalized number of polar beads.

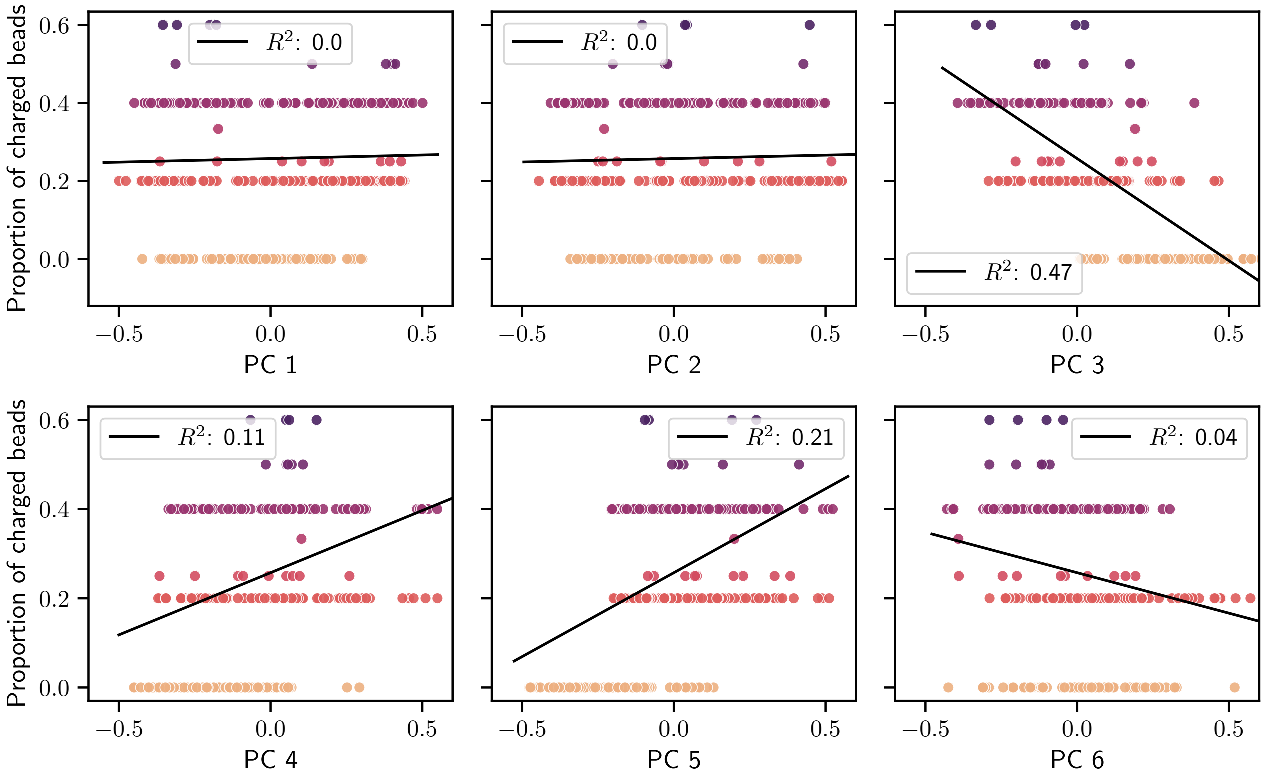

Figure S9: Cross-correlation of the first six principal components and the normalized number of charged beads per solute (bead type Q0). Again, the strongest correlation is found with PC3, and also, to a lesser extend, in both PC5 and PC4. Color gradient: the normalized number of charged beads.

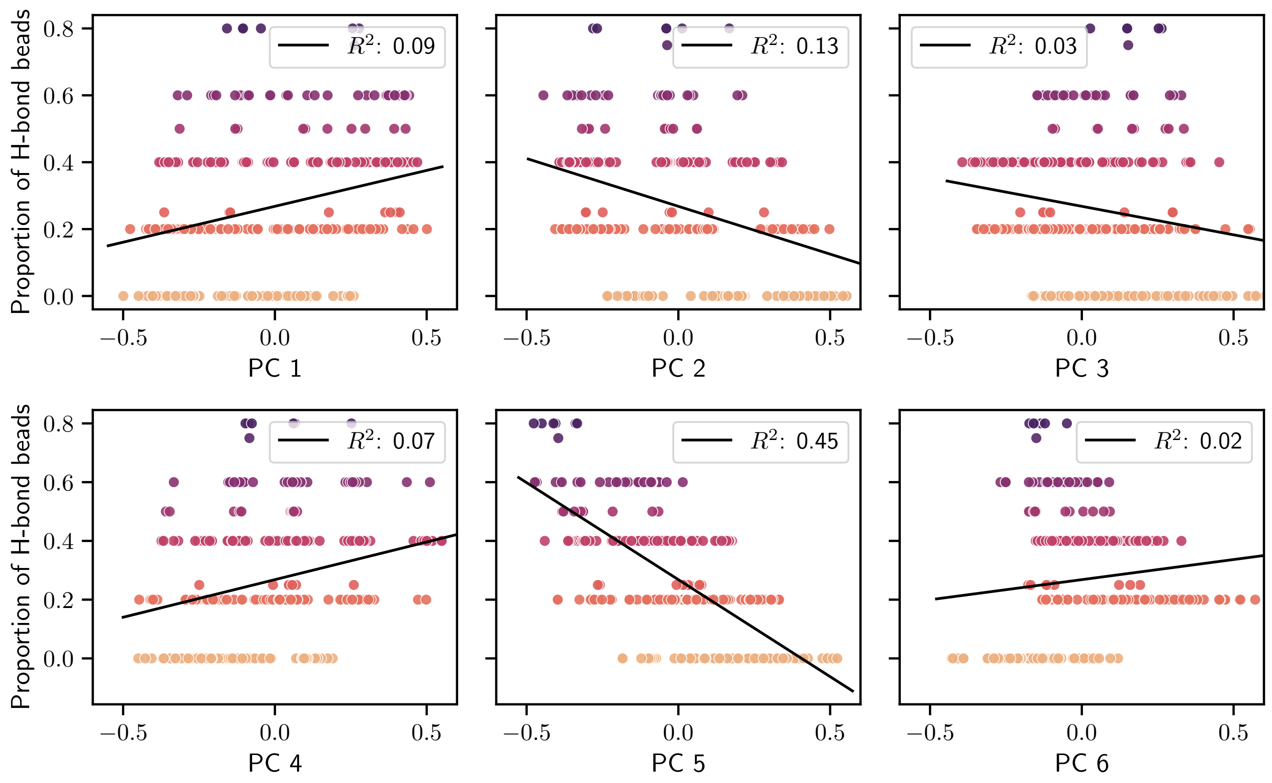

Figure S10: Cross-correlation of the first six principal components and the ratio of hydrogen-bonding beads per solute (bead type T3). Here, we find the strongest correlation to PC5, with a weaker correlation also visible with PC2. Color gradient: the normalized number of hydrogen-bonding beads.

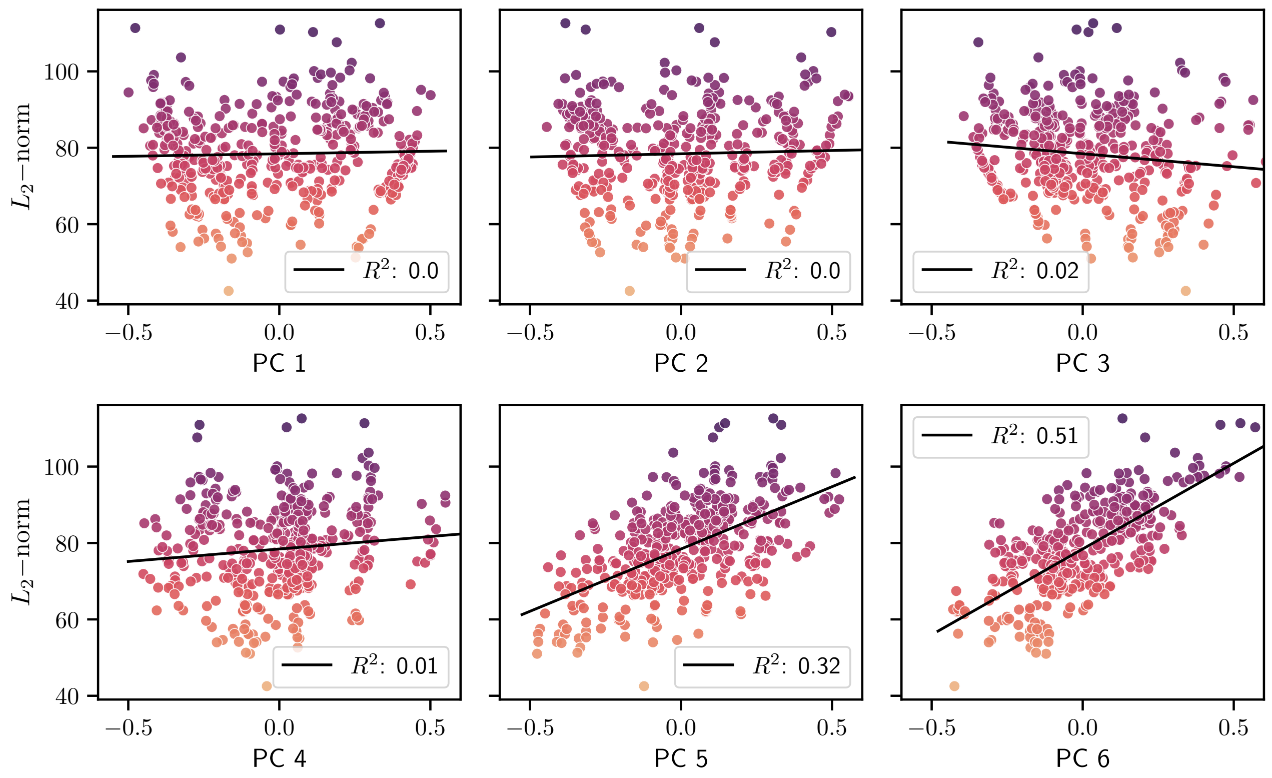

Figure S11: Cross-correlation of the first six principal components and the Euclidian distance (L_2-norm) of the $\Delta\langle\mathcal{X}\rangle$, quantifying the difference in detected interactions in the CL and PG environments. The L_2-norm is mainly correlated with PC6, but also to PC5. Color gradient: L_2-norm.

5 Correlations between the first six principal components and the many-body interactions

PCA as implemented by `scikit-learn` is based on the correlation matrix of the standardized and centered original variables. Scaling the PC loadings by the square root of their corresponding eigenvalue,

$$l_{k,\text{scaled}} = v_k * \sqrt{\lambda_k}, \tag{S2}$$

in this case shows the correlation between the original variables and the respective PC by combining directional information and magnitude of the variance. We analyze the $l_{k,\text{scaled}}$ to identify interactions with high impact on each of the six analyzed PCs.

The scaled loadings are sorted by descending magnitude, up to an absolute value of 1.0. We colored the individual bars by the $\Delta G_{\text{w}\rightarrow\text{Ol}}$ of the corresponding many-body interaction to support the visual interpretability of the figures.

The presence of the beads representing the lipid headgroups (Nda or P4) in dominant $l_{k,\text{scaled}}$ shows the prominent role of the latter in many-body interactions. Noteworthy here is the perfect separation of interactions involving either CL or PG but sharing all other bead types by opposite signs, as visible by the presence of the Nda or the P4 beads in the many-body interactions.

Attention is drawn to the PCs with clearly identifiable cross-correlations to descriptors connected to CL selectivity, PC3, PC5 and PC6. With PC3 (Figure S12c) and PC5 (S13b), interactions differing only in the presence of the CL vs the PG headgroup bead (Nda vs P4) have approximately equal absolute values of $l_{k,\text{scaled}}$ with opposite signs, showing a clear pairwise negative correlation. The interactions with the highest absolute values of $l_{3,\text{scaled}}$ (Figure S12c) predominantly involve charges (Q0), hydrogen bonding (T3) and apolar beads (T4). The $l_{5,\text{scaled}}$ (S13b) show that interactions involving the hydrogen-bonding bead type (T3) are negatively correlated to interactions involving no hydrogen bonding, highlighting the correlation of PC5 to the number of hydrogen bonds per solute identified by direct cross-correlation, Figure 3 (b). In the case of the $l_{6,\text{scaled}}$ (Figure S13c), the negative correlation between interactions involving the CL vs the PG headgroup beads is less pronounced than in in the previous cases. By direct cross-correlation, PC6 was linked to the L_2-norm as a measure of the difference in the encountered interactions and their frequency between a solute and the CL versus the PG environments, providing a possible explanation for this effect. The most dominant correlations between the many-body interactions and PC1, PC2 and PC4 (Figure S12a, b and S13a) are included for completeness.

High absolute values of $l_{3,\text{scaled}}$ are further investigated as an indicator of the relevance of specific two- and three-body interactions for the desired effect of increased CL selectivity.

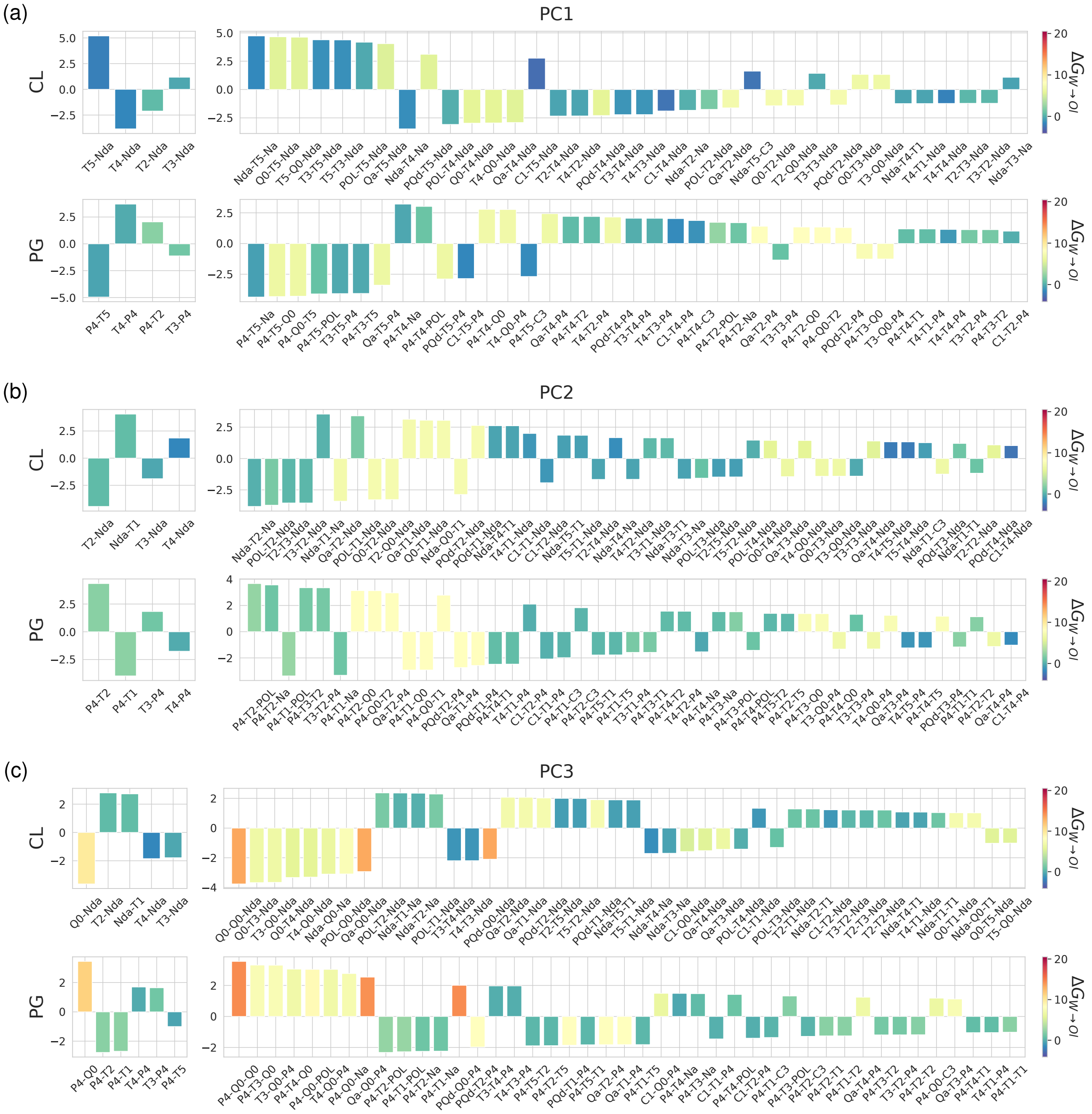

Figure S12: Scaled loadings of the many-body interactions of PC1, PC2 and PC3, sorted by absolute value. For each principal component, the two-body interactions are shown on the left and the three-body interactions in the right column. The individual bars are colored by the $\Delta G_{\mathrm{W}\to\mathrm{Ol}}$ of the corresponding many-body interaction to enhance visual separation. The interactions involving the CL headgroup bead Nda are isolated in the top panel from the interactions involving the PG headgroup bead P4 in the bottom panel.

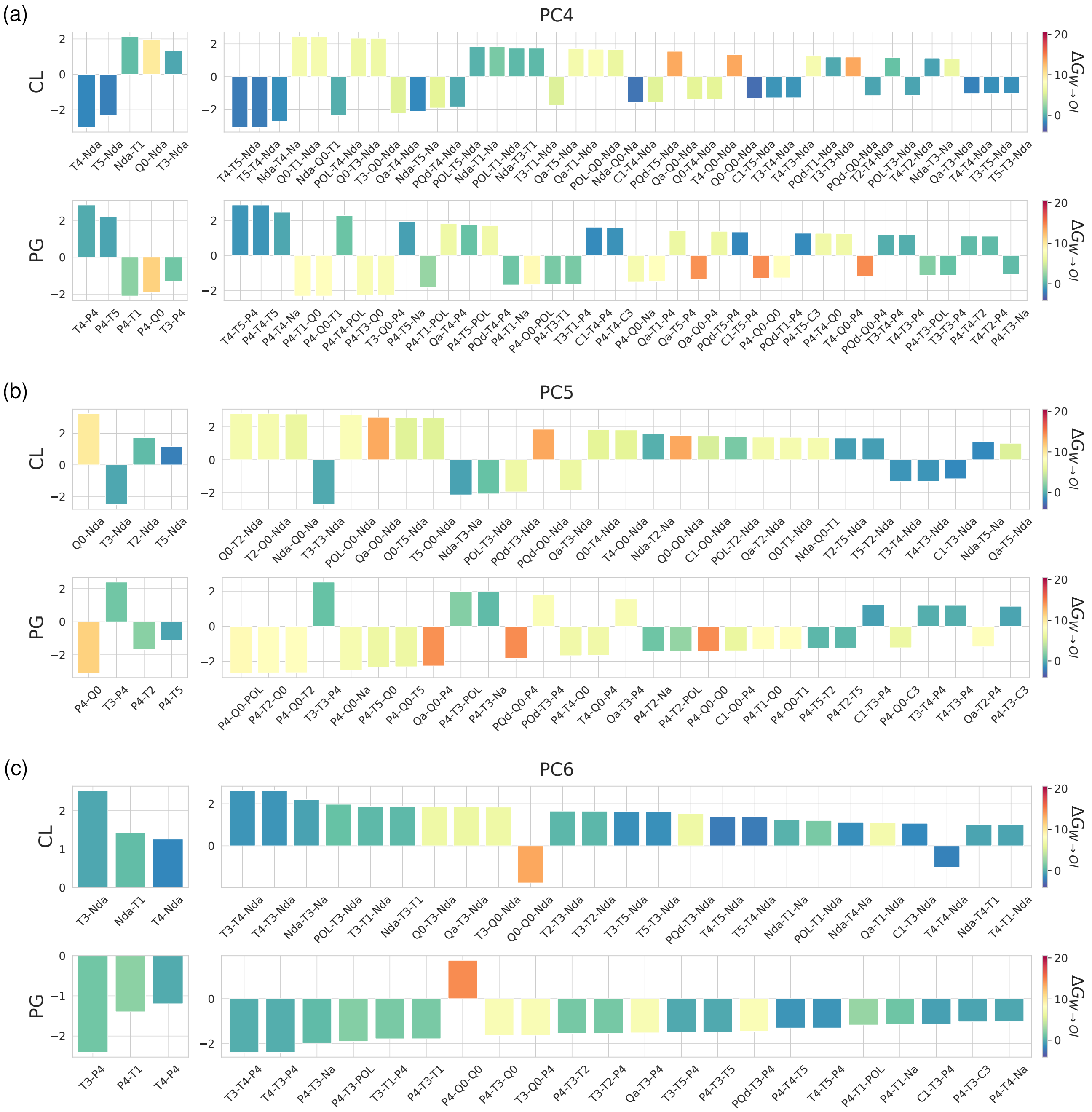

Figure S13: Scaled loadings of the many-body interactions of PC4, PC5 and PC6, sorted by absolute value. The two-body and three-body interactions are again separated between left and right columns, and interactions involving CL and PG are again shown in the top row and bottom row of each plot, respectively. The bars are colored in the same fashion as in Figure S12

6 Interaction graphs

We investigate the scaled loadings $l_{k,\text{scaled}}$ (Equation (S2)) with high absolute values to more clearly identify the interactions that are most relevant for the respective PC. In Figure S14, we illustrate the 33 highest $l_{3,\text{scaled}}$ with negative signs, linked to increased CL selectivity in section 5. Here, we make no distinction between two-body and three-body interactions. The interactions are sorted by the lipid headgroup beads (Nda and P4). The same bead types participating in multiple different interactions are grouped.

We included the 50 values of $l_{3,\text{scaled}}$ with maximum absolute values to represent the prevalence

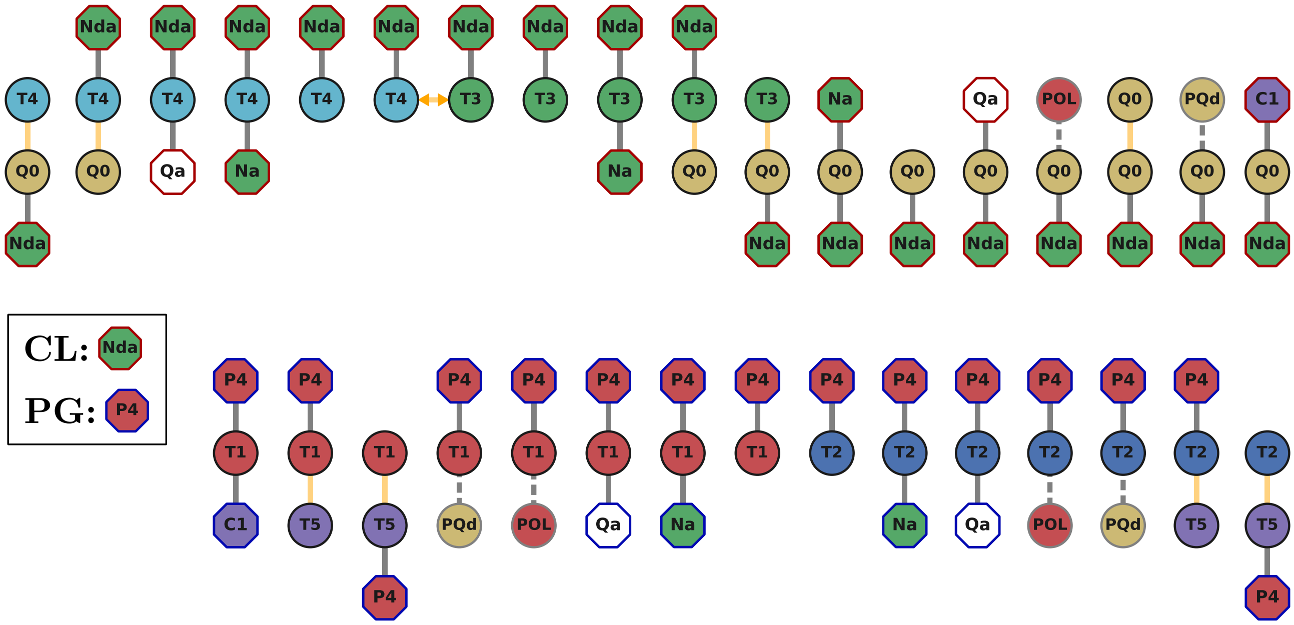

Figure S14: Illustration of the two- and three-body interactions included in Figure 4 in the main text, only the 33 values of $l_{3,\text{scaled}}$ with largest absolute values are represented here. The interactions are sorted by presence of the respective lipid headgroups and arranged to highlight recurring patterns of the same bead types or bead types with similar properties interacting. The orange bonds represent intramolecular bonds of the solutes, the two-sided orange arrow indicates the presence of both Nda-T4-T3 and Nda-T3-T4. The dashed lines indicate bonds between solute and solvent representations.

of the observed interactions, as well as the most common interaction partners in the form of graph representations (Figures S15 to S20). We can use these interaction graphs to gain more detailed information about the interactions associated with the respective principal components. Furthermore, we are able to create possible three-dimensional CG structures maximizing the respective property associated with the corresponding PC. As visual aides, the solute bead types are shown as circles with black borders and the solvent bead types as circles with grey borders. The lipid bead types have an octagonal shape and a red border for CL and a blue border for PG. The orange edges indicate intramolecular interactions between two beads belonging to the set used for representing the solutes, gray edges show intermolecular interactions between solute beads and lipid beads, and the dashed edges indicate interactions with a solvent bead type. We only included the bead types that we encountered in the 50 largest values of $l_{k,\text{scaled}}$ for each PC.
Overall, the graphs confirm the meaning of the lower-dimensional variables associated with the PCs and the significance of specific many-body interactions shown in Figures S12 and S13. A quasi-three-dimensional interpretation of the information we gleaned from the interactions is shown in Figure 5.

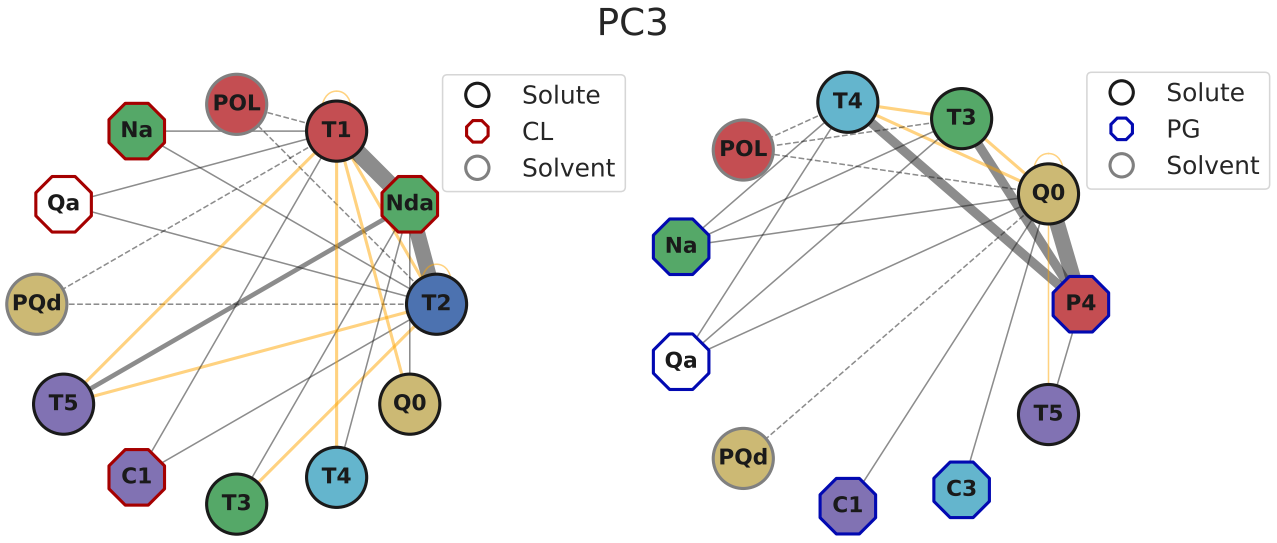

Figure S15: Graph visualization of the two-body and three-body interactions with the strongest correlation to PC3. Here, only the subset of the 50 highest values of $l_{3,\mathrm{scaled}}$ with positive signs is shown, interactions involving the CL headgroup bead Nda in the left graph, interactions involving the PG headgroup bead P4 on the right. The width of the edges represents the number of times an interaction between two beads was encountered in this subset. Edges highlighted in orange show interactions between solute beads, and dashed edges represent interactions with beads used to model water or sodium ions. We found PC3 to be correlated to the selectivity $\Delta\Delta G$, the positive values of $l_{3,\mathrm{scaled}}$ therefore indicate spacial solute-lipid interactions with potential negative correlation to CL selectivity. The graphs generated from the values of $l_{3,\mathrm{scaled}}$ with negative signs, correlated to increased CL selectivity, are shown in the main text, Figure 5.

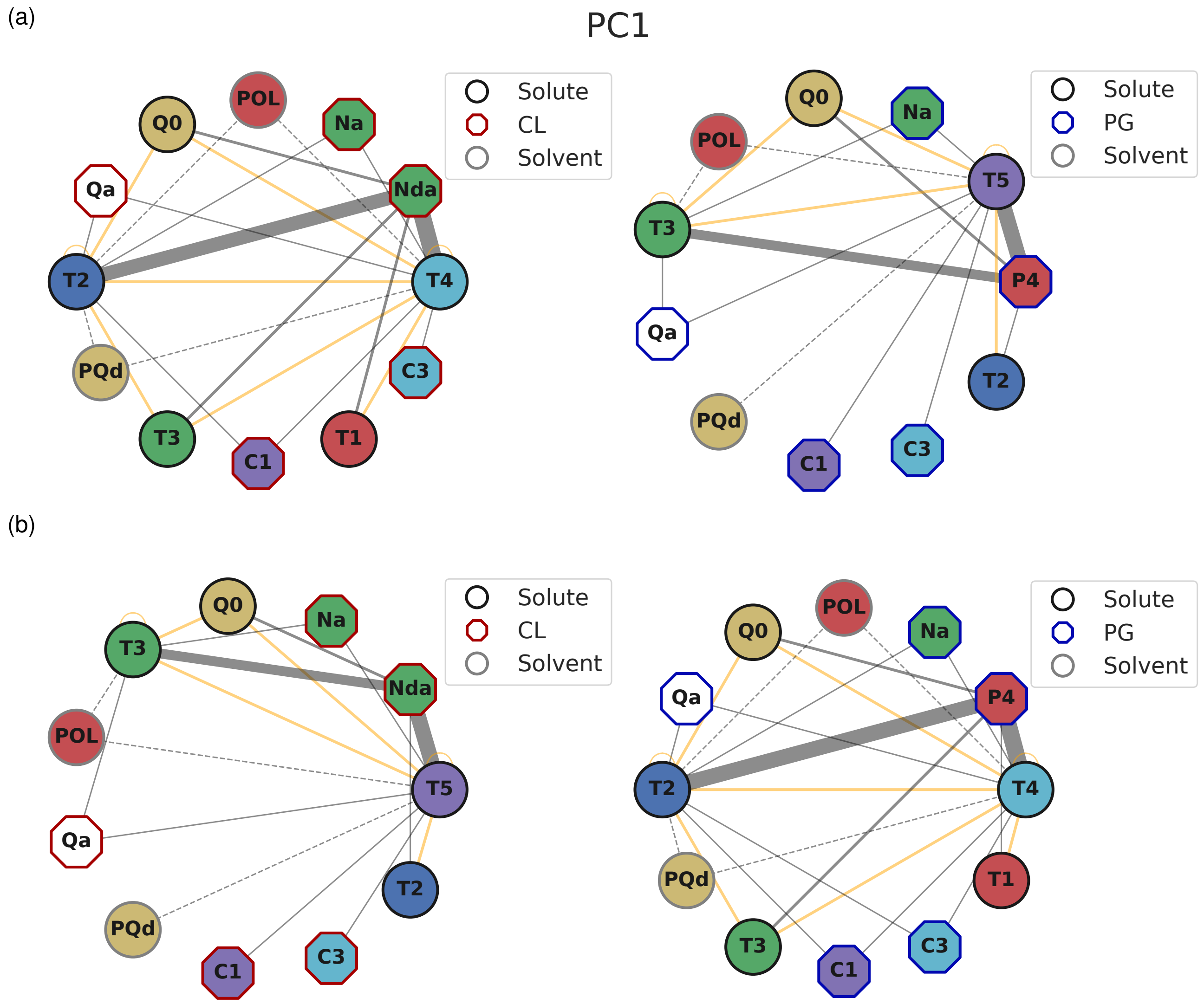

Figure S16: Graph visualization of the most common interactions generated by analyzing the two-body and three-body interactions with the maximum absolute values for $l_{1,\mathrm{scaled}}$. Panel (a) shows the subset of the 50 highest values of $l_{1,\mathrm{scaled}}$ with negative signs, interactions involving the CL headgroup bead Nda in the left graph, interactions involving the PG headgroup bead P4 is shown on the right. The color, style and weight of the edges have the same significance as in Figure S15. The graphs shown in panel (b) are generated from the 50 highest values of $l_{1,\mathrm{scaled}}$ with positive signs. PC1 showed a comparatively weaker interaction with the number of polar beads in a solute (Figure S8), and from the graphs we can see that the weaker polar bead T2 and the less hydrophobic bead T4 seem to have a more prominent role in the formation of polar interactions between solutes and CL. In comparison, the stronger polar T1 bead and the most hydrophobic T5 bead seem to favor PG. Again, the headgroup beads of both lipids are predominantly represented in solute-lipid-interactions. Interestingly, we also observe a high frequency of interactions between the hydrophobic beads and the lipid headgroups, despite not seeing a correlation between PC1 and hydrophobicity.

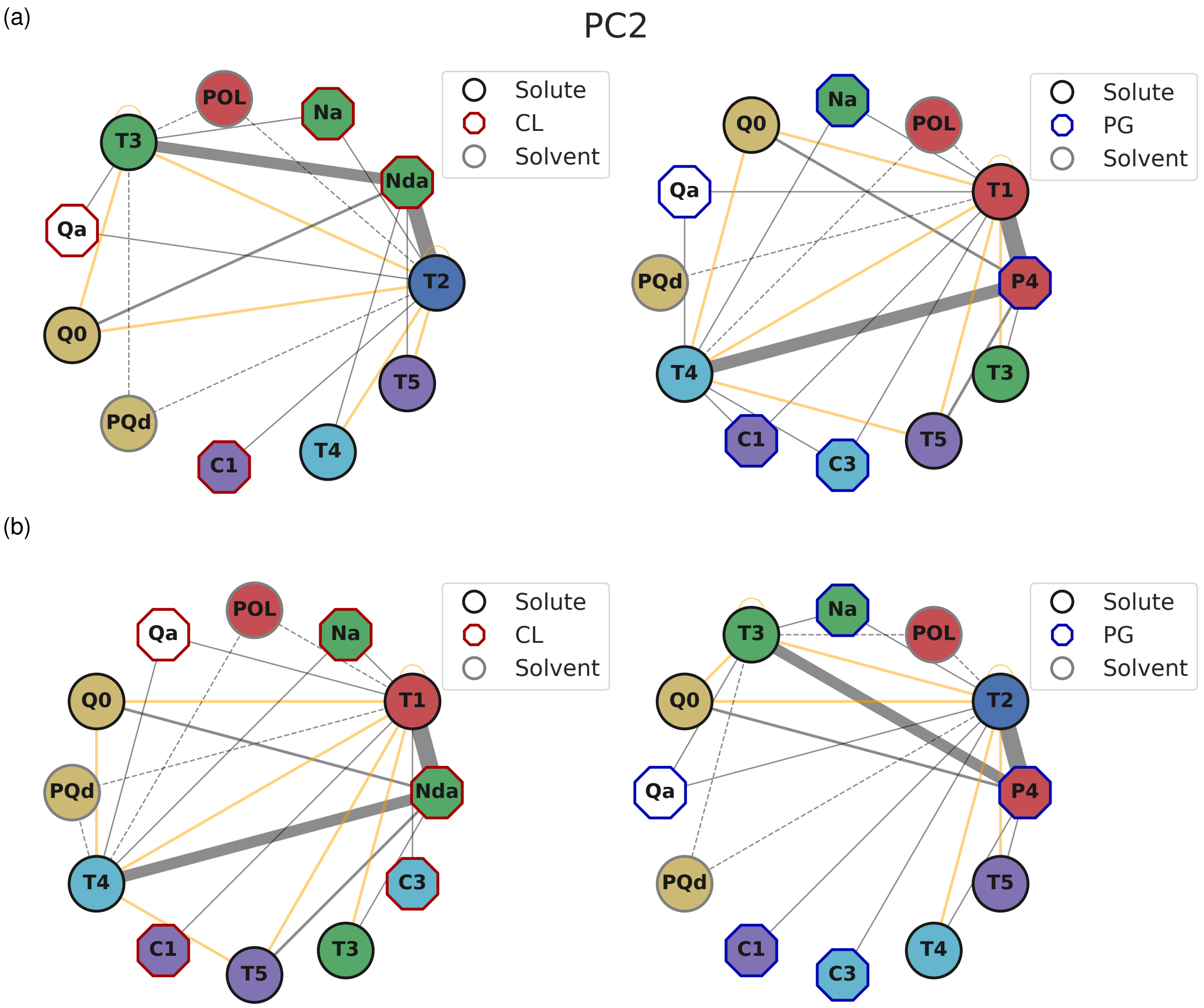

Figure S17: Graph visualization of the most common interactions generated by analyzing the two-body and three-body interactions with the maximum absolute values of $l_{2,\text{scaled}}$. Panel (a) shows the subset of the 50 highest values of $l_{2,\text{scaled}}$ with negative signs, interactions involving the CL headgroup bead Nda in the left graph, interactions involving the PG headgroup bead P4 is shown on the right. The color, style and weight of the edges have the same significance as in Figure S15. The graphs shown in panel (b) are generated from the 50 highest values of $l_{2,\text{scaled}}$ with positive signs. We found PC2 weakly correlated to the number of hydrogen-bonding sites (Figure S10), which is reflected in the frequency of the interactions between the T3 and the CL-Nda bead. We also observe a high frequency of interactions between the polar beads and the lipid headgroups (T2-Nda for CL, T1-P4 for PG), although we did not see a correlation between PC2 and the ratio of polar beads in the solutes.

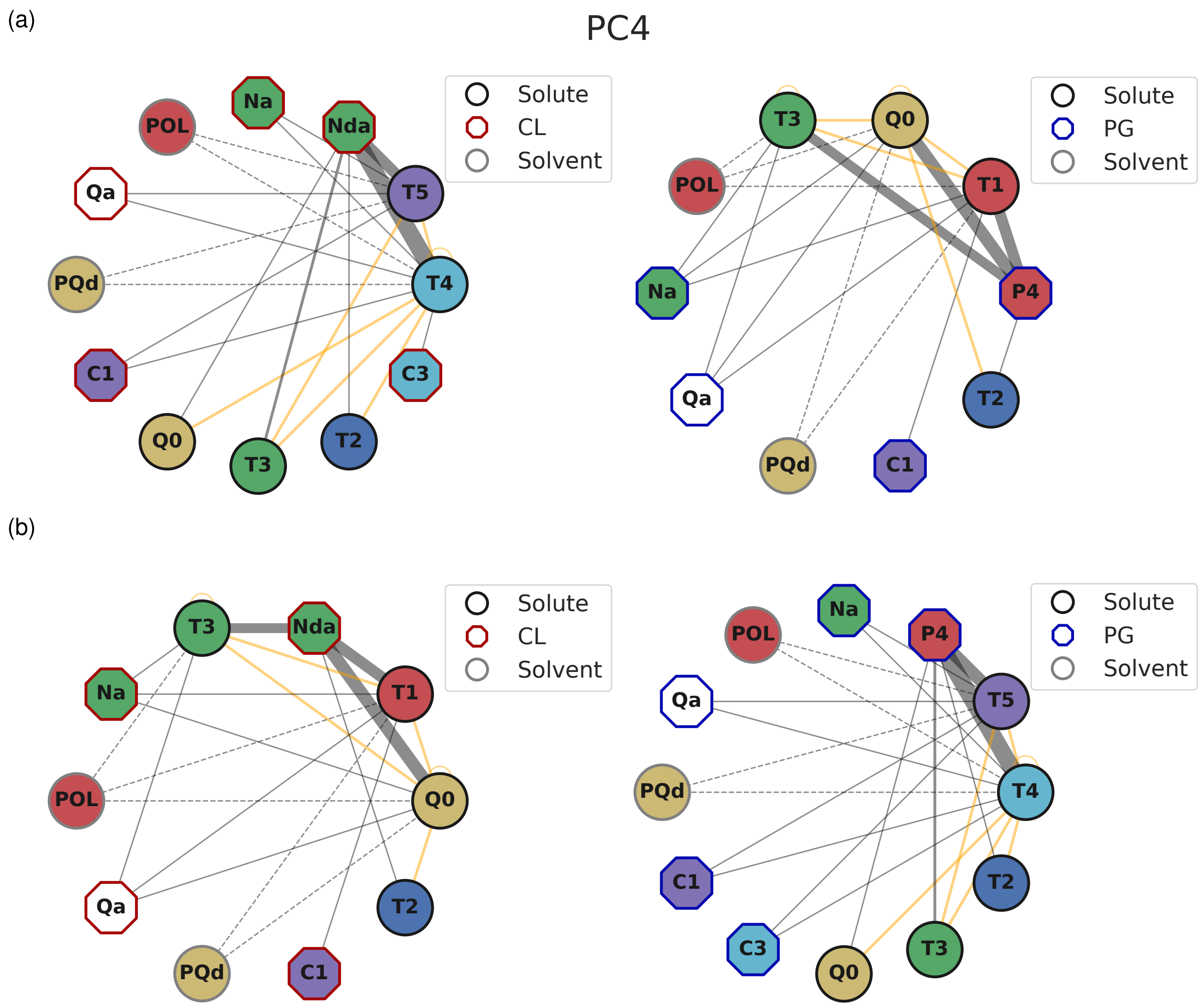

Figure S18: Graph visualization of the most common interactions generated by analyzing the two-body and three-body interactions with the maximum absolute values of $l_{4,\text{scaled}}$. Panel (a) shows the subset of the 50 highest values of $l_{4,\text{scaled}}$ with negative signs, interactions involving the CL headgroup bead Nda in the left graph, interactions involving the PG headgroup bead P4 are shown on the right. The color, style and weight of the edges have the same significance as in Figure S15. The graphs shown in panel (b) are generated from the 50 highest values of $l_{4,\text{scaled}}$ with positive signs. We found PC4 to be correlated to the hydrophobicity $\Delta G_{\text{W}\rightarrow\text{Ol}}$ (Figure S7), which is reflected (a) for CL and (b) for PG. The correlation of PC4 to the ratio of charges per solute beads (Figure S9) leads to the graph in (b) for CL and (a) for PG.

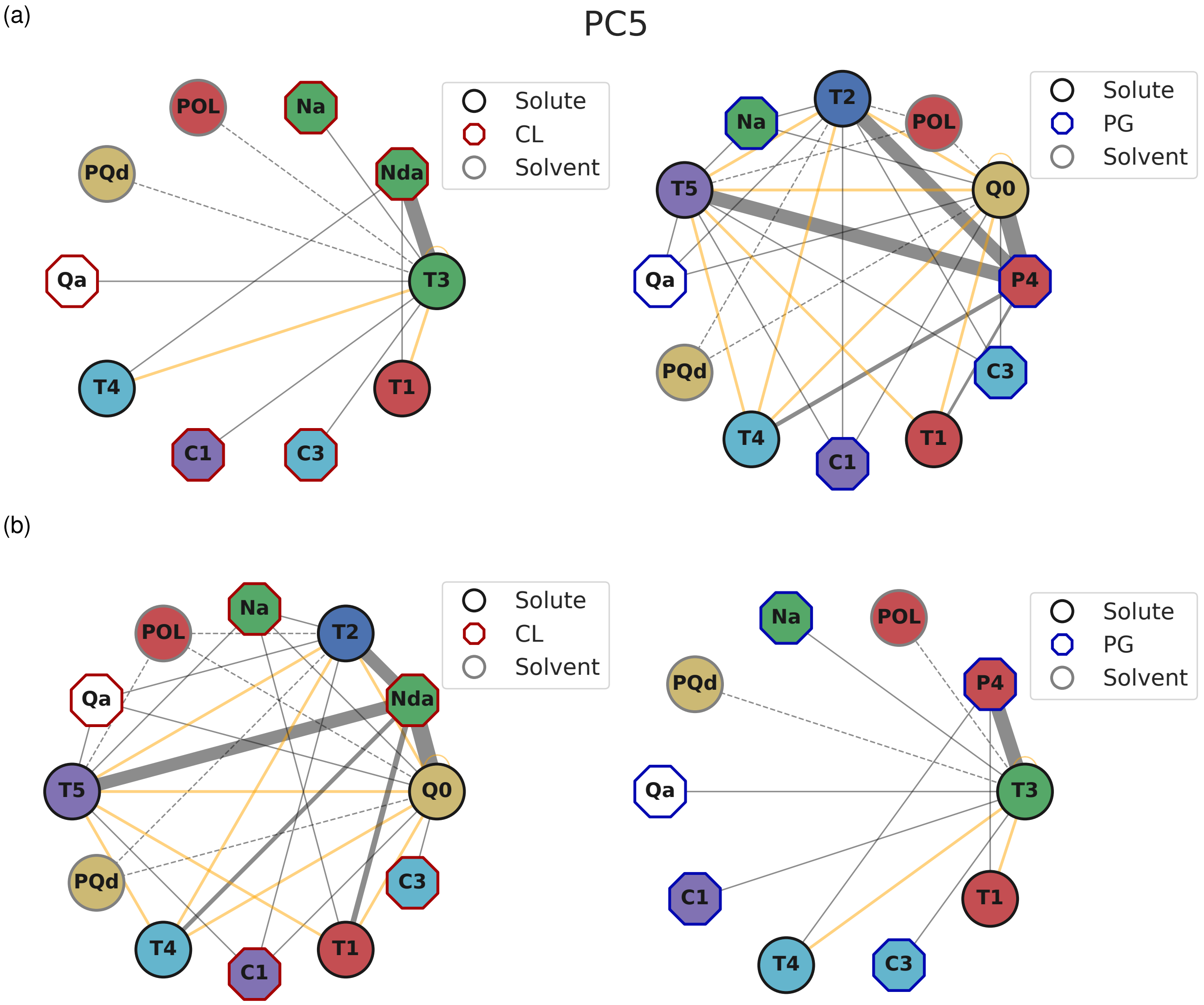

Figure S19: Graphs generated from the values of $l_{5,\text{scaled}}$, which highlights the formation of hydrogen bonds between a solute and the CL headgroup. The graphs in (a) reflect the 50 highest values of $l_{5,\text{scaled}}$ with negative signs, the equivalent with positive signs are represented in (b), and again the interactions involving CL and PG are shown in the left and right columns, respectively. The color, style and weight of the edges have the same significance as in Figure S15.

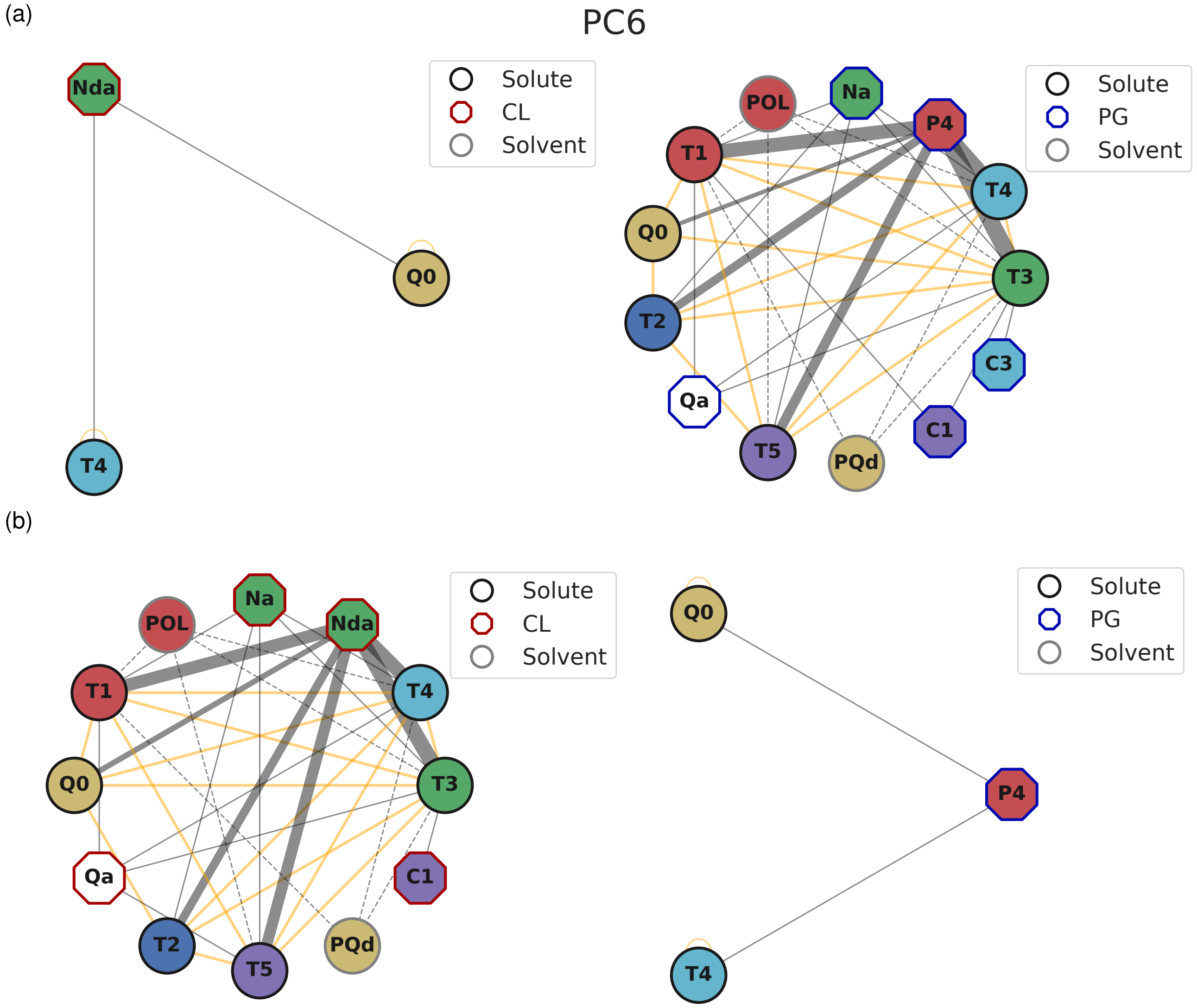

Figure S20: Graph visualization of the most common two-body and three-body interactions with the maximum absolute values of $l_{6,\text{scaled}}$. Panel (a) shows the subset of the 50 highest values of $l_{6,\text{scaled}}$ with negative signs, interactions involving the CL headgroup bead Nda in the left graph, interactions involving the PG headgroup bead P4 is shown on the right. The color, style and weight of the edges have the same significance as in Figure S15. The graphs shown in panel (b) are generated from the 50 highest values of $l_{6,\text{scaled}}$ with positive signs. PC6 is most strongly correlated to the L^2-norm as a measure of the difference between interactions observed in the CL and the PG environments. Interestingly, here the charged and mildly hydrophobic bead types (Q0 and T4) are singled out despite interacting only comparatively infrequently with the lipid headgroups. The significance with respect to the difference in observed interactions is not immediately clear.

7 Biplots of the main six principal components and their corresponding eigenvectors

Biplots allow the visualization of the structures of the samples in a data set relative to the observables.[10] We include biplots of all pairwise combinations of the main six principal components, colored by the same descriptors we identified as associated with the principal components in Figures S6 to S11. To increase visibility, we always include a plot with the largest eigenvalue-coefficients of two-body interactions and the same plot including the largest eigenvalue-coefficients of the three-body interactions. The correlations of multiple PCs to the same metric becomes increasingly clear here in the separation of the color gradient in biplots fo the respective PCs. By following the color gradients, we are able to determine which direction of a PC points to positive or negative correlation with the respective metric. In the cases of multi-correlation, the largest loadings also tend to correspond to the same two- or three-body interactions and point into the same direction along the color gradients. It is also common for different coefficients pointing into the same or very similar directions, leading to overlapping of their labels in the plots. This again shows that the interactions we observe in our data set are themselves highly correlated.

We do not see clear separation between groups of samples in most cases, which can also be attributed to the highly inter-correlated nature of our observables. Many-body interactions involving the same or similar bead types will inevitably be correlated, and the differences in the lipid models are limited to the headgroup bead and the overall number of beads.

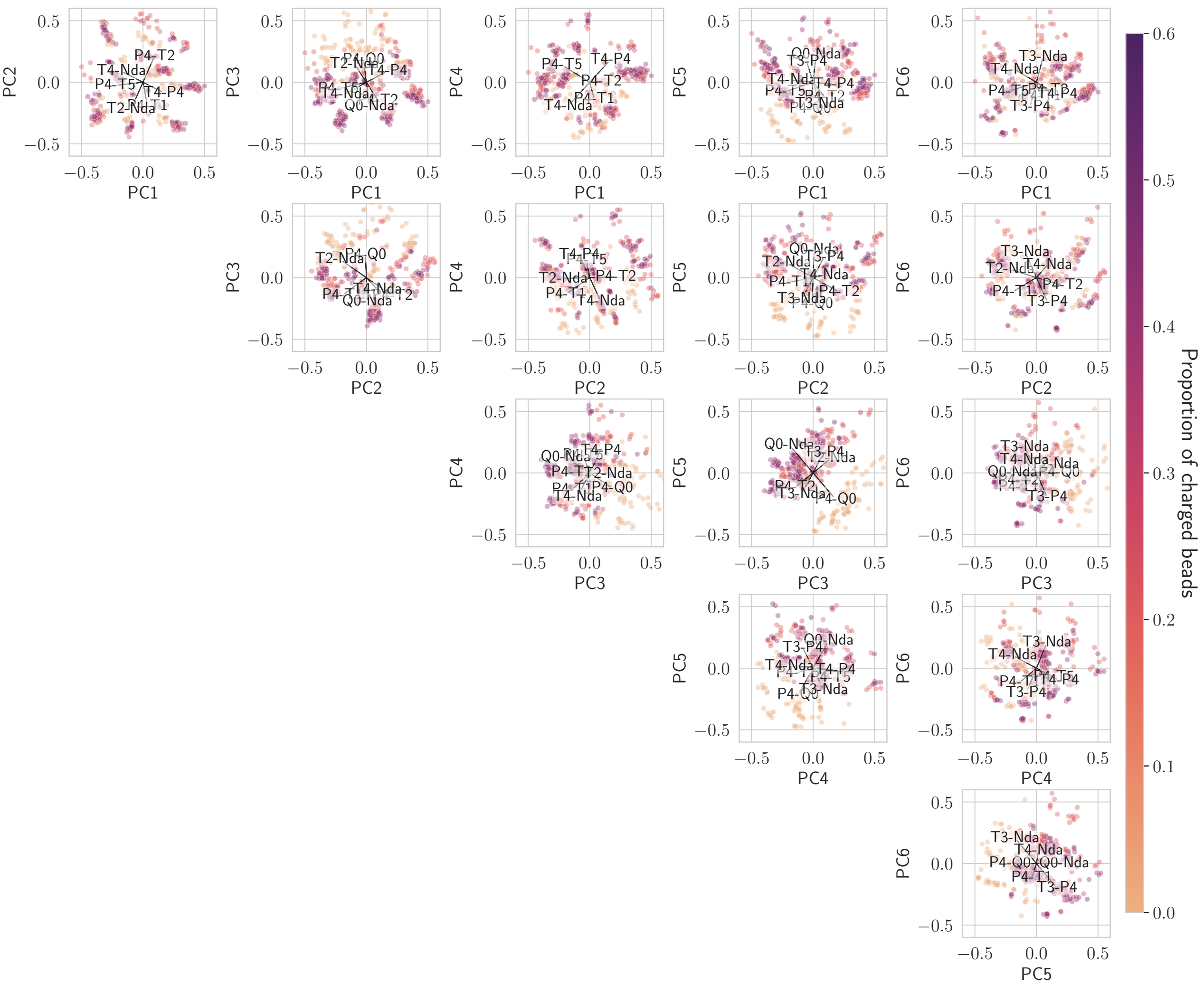

Figure S21: Biplots of all pairs of principal components with the largest loadings corresponding to two-body interactions. The samples are colored by the ratio of charged beads (Q0) per solute. PC3, PC5 and PC4 are correlated to net charge (Figure S9), reflected in the color separation of the corresponding biplots. Most notably, in the plot showing PC3 and PC5, the color gradient is split along the separation between two groups of solutes. In this case, PCA was able to separate neutral vs. charged compounds.

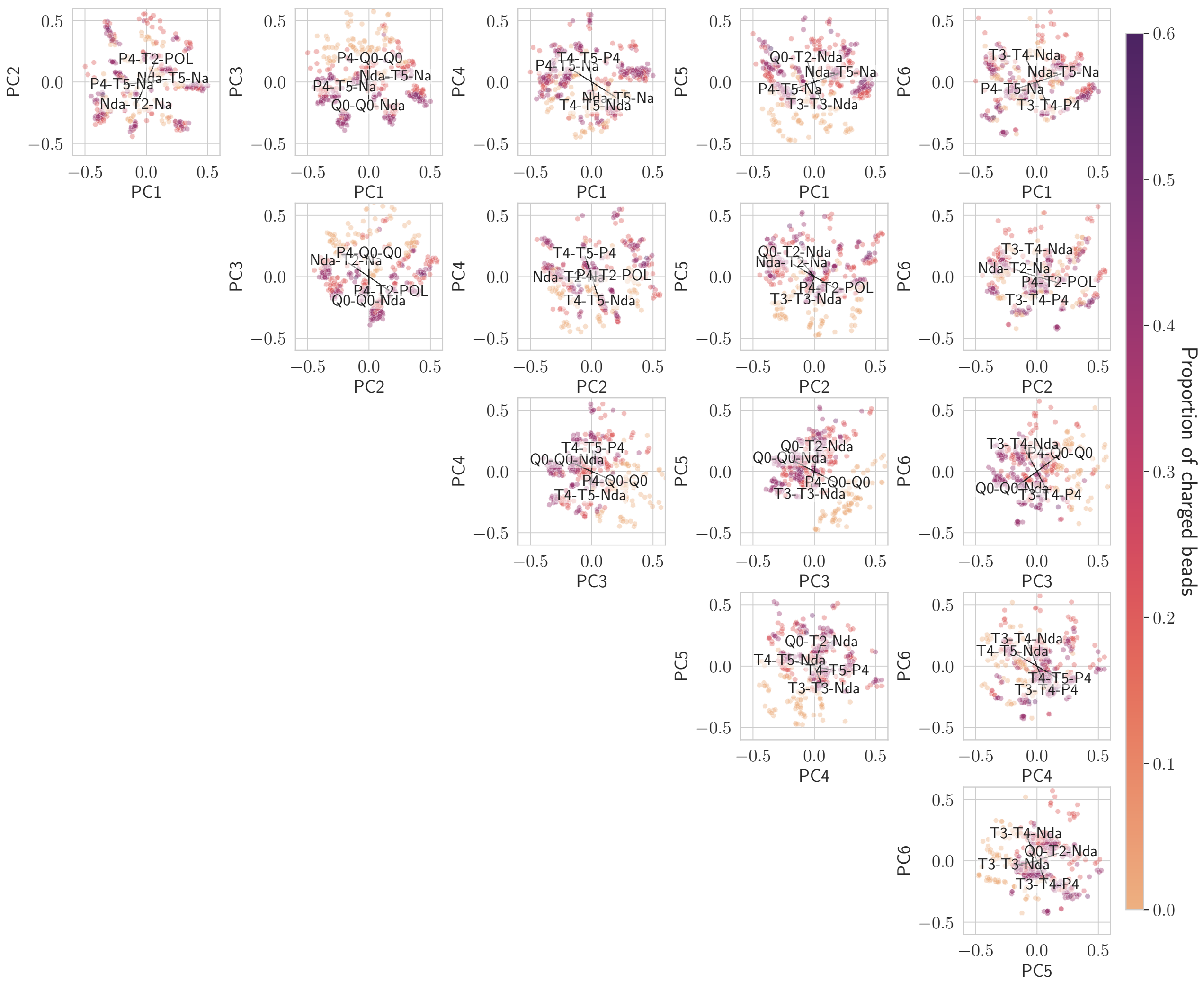

Figure S22: Biplots of all pairs of principal components with the largest loadings corresponding to three-body interactions. The samples are colored by the ratio of charged beads (Q0) per solute. PC3, PC5 and PC4 are correlated to net charge (Figure S9), reflected in the color separation of the corresponding biplots. The clear separation between charged and neutral compounds is also highlighted by the direction of the loadings resembling three-body interactions including charges.

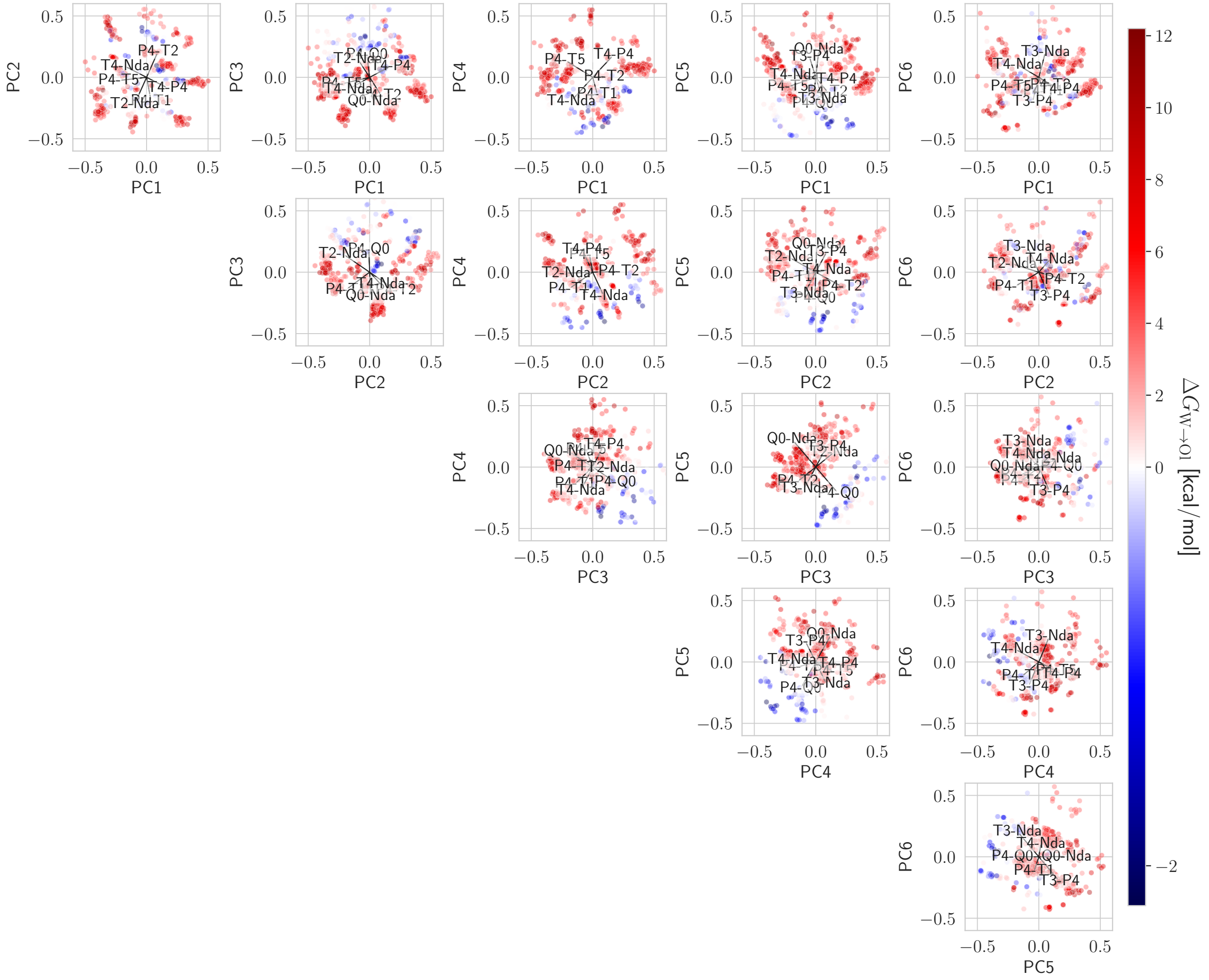

Figure S23: Biplots of all pairs of principal components with the largest six loadings corresponding to two-body interactions. The samples are colored by the average hydrophobicity $\Delta G_{\mathrm{W}\to\mathrm{Ol}}$. We have found PC3 to be correlated to hydrophobicity as well as PC4 and PC5 (Figure S7). Here, the multi-correlation is visible even more clearly in the clean color separation of the samples in plots involving pairs of PC3, PC4 and PC5.

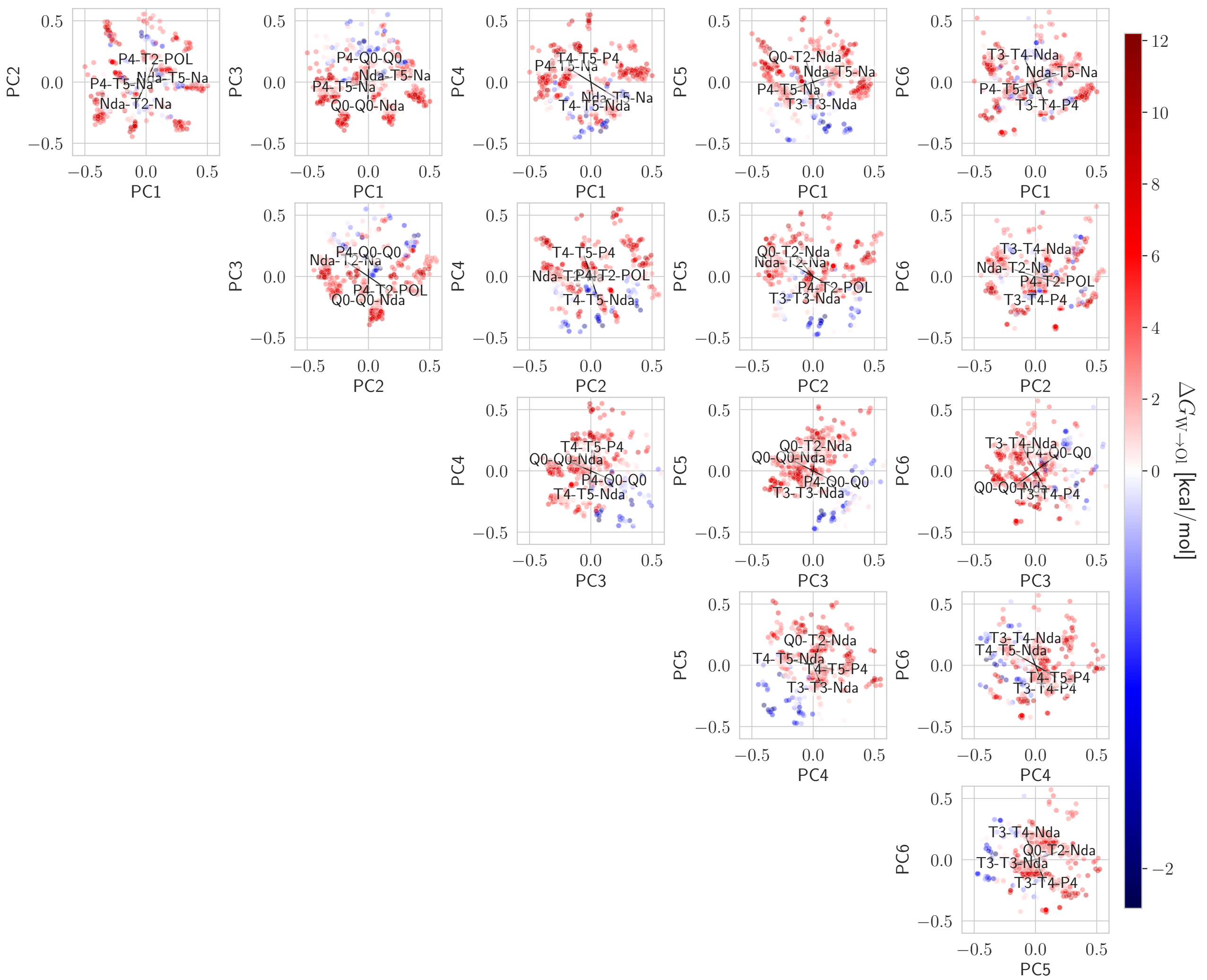

Figure S24: Biplots of all pairs of principal components with the largest four loadings corresponding to three-body interactions. The samples are colored by the average hydrophobicity $\Delta G_{\mathrm{W}\rightarrow\mathrm{Ol}}$.

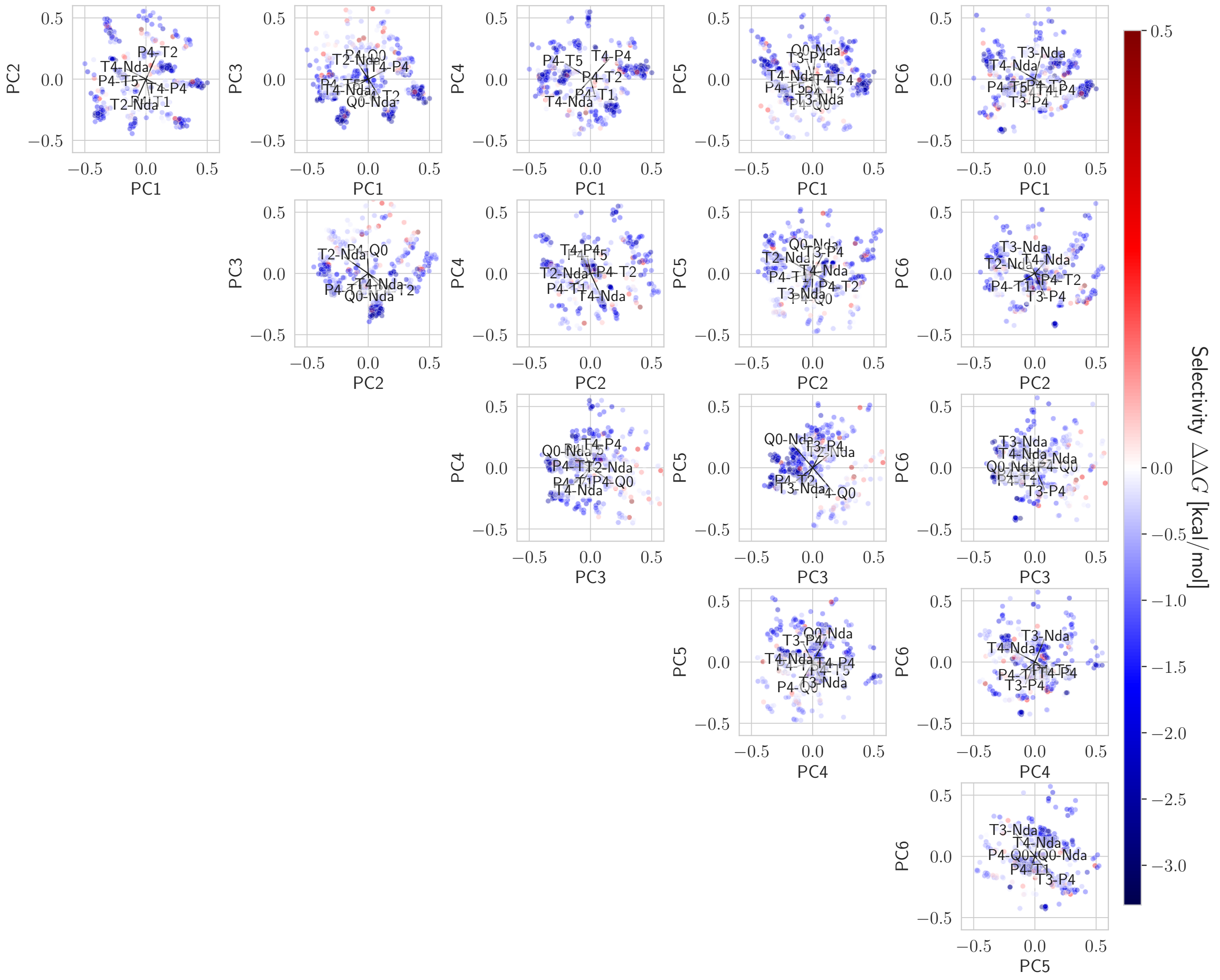

Figure S25: Biplots of all pairs of principal components with the largest six loadings corresponding to two-body interactions. The samples are colored by the selectivity $\Delta\Delta G$. Only PC3 can be correlated to selectivity, which leads to only imperfect color separation.

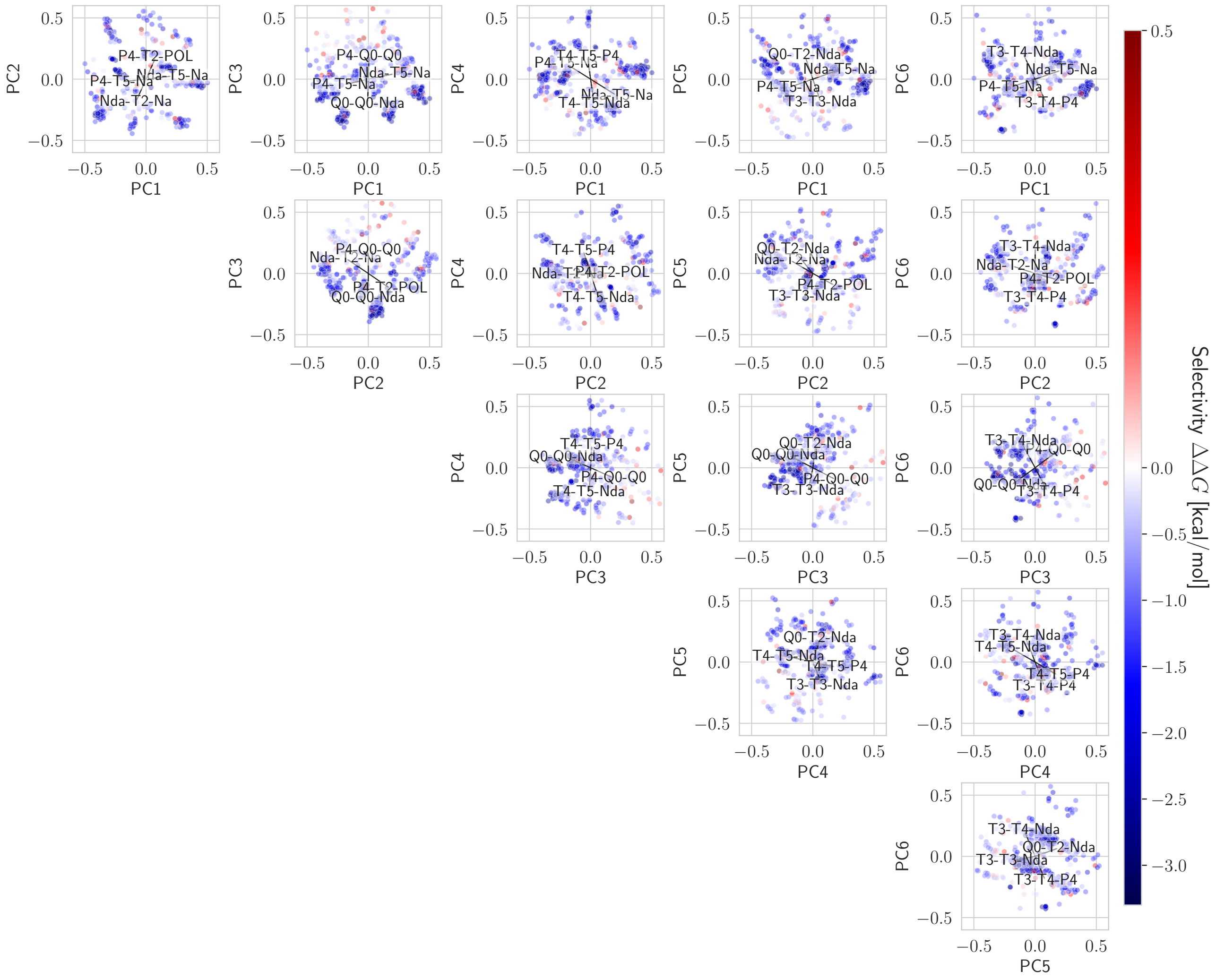

Figure S26: Biplots of all pairs of principal components with the largest four loadings corresponding to three-body interactions. The samples are colored by the selectivity $\Delta\Delta G$. Only PC3 can be correlated to selectivity, which leads to only imperfect color separation.

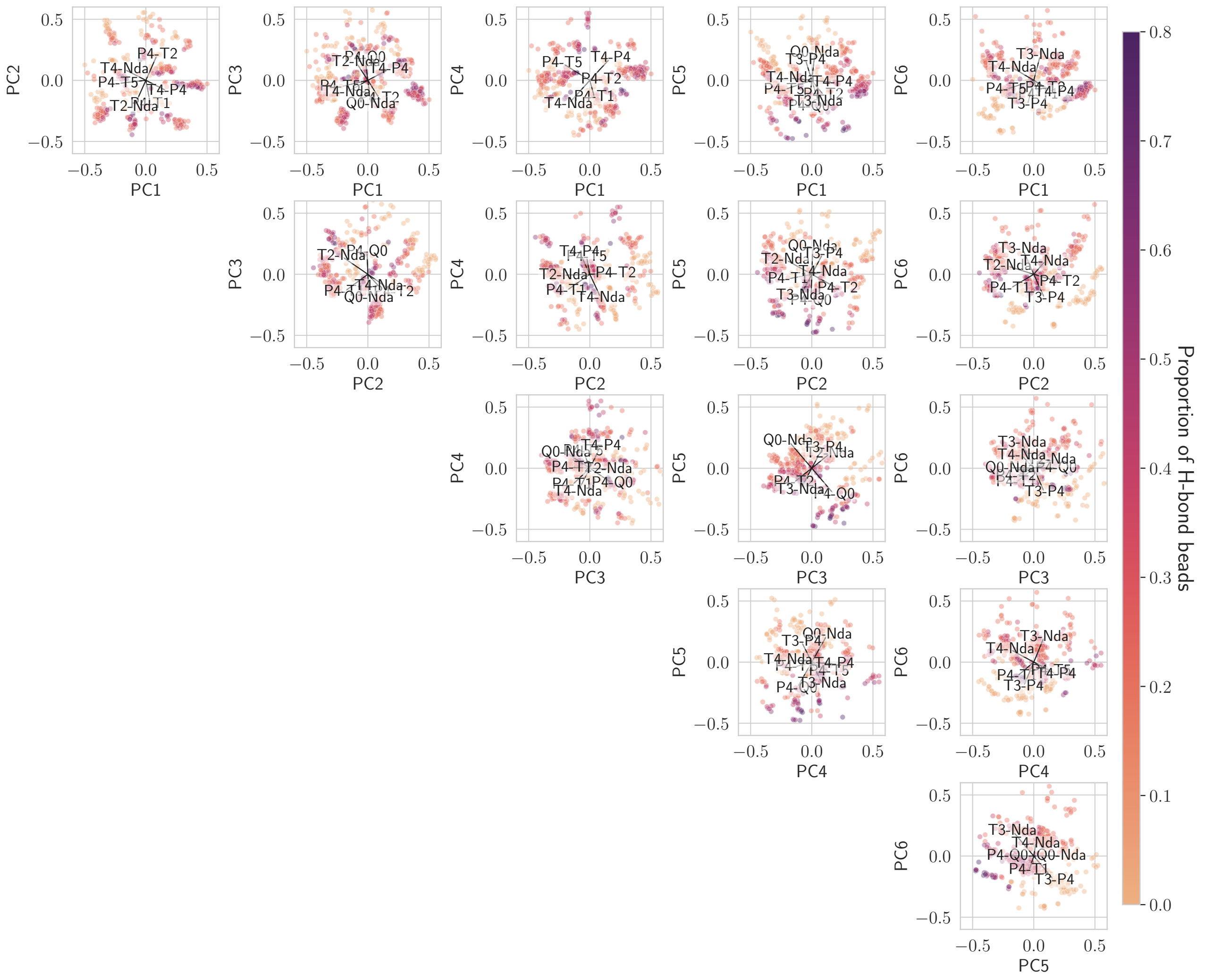

Figure S27: Biplots of all pairs of principal components with the largest six loadings corresponding to two-body interactions. The samples are colored by the ratio of hydrogen-bonding beads (T3) per solute. PC5 and PC2 are correlated to hydrogen bonding (Figure S10), reflected in the color separation of the corresponding biplot.

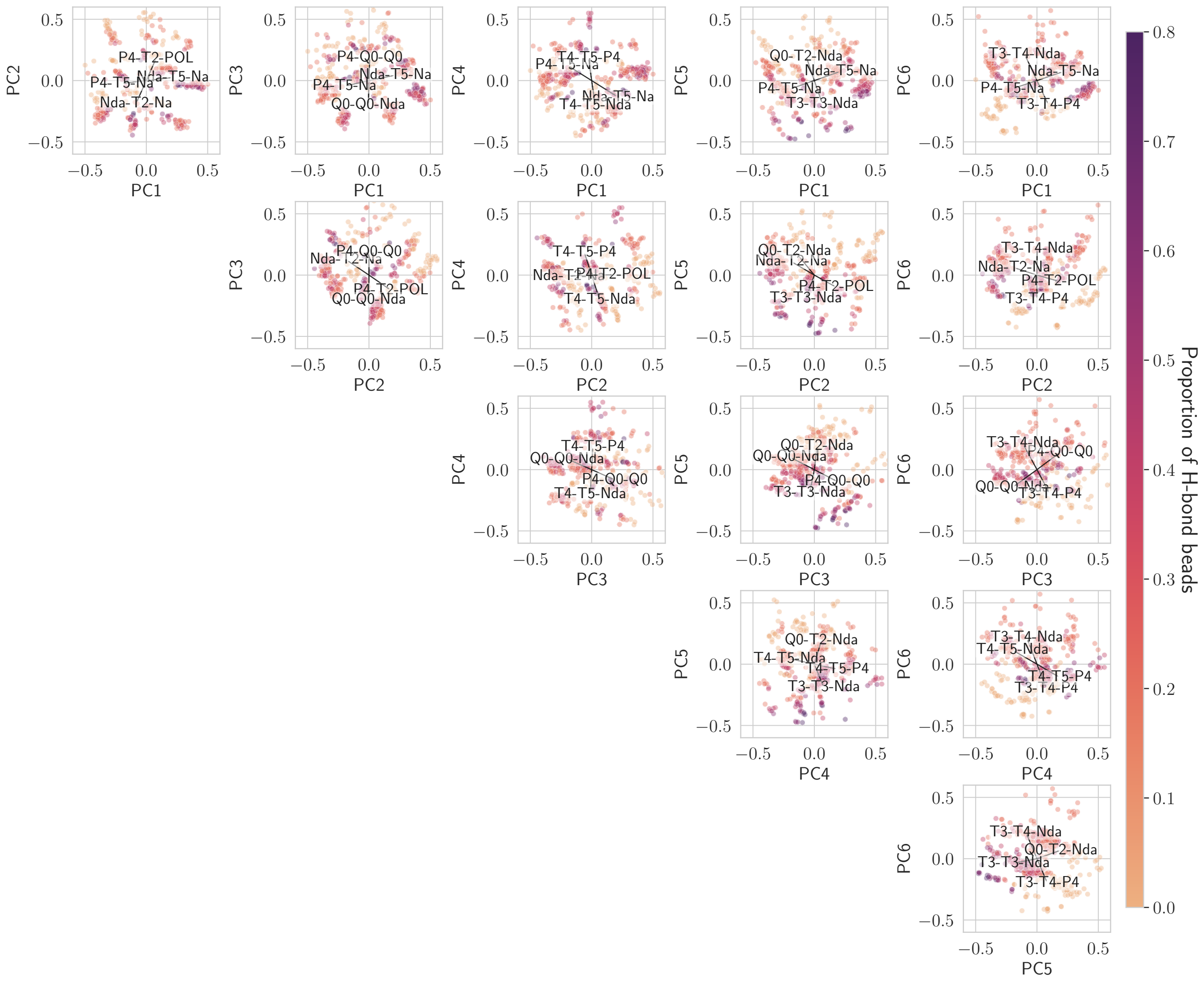

Figure S28: Biplots of all pairs of principal components with the largest four loadings corresponding to three-body interactions. The samples are colored by the ratio of hydrogen-bonding beads (T3) per solute. PC5 and PC2 are correlated to hydrogen bonding (Figure S10), reflected in the color separation of the corresponding biplot.

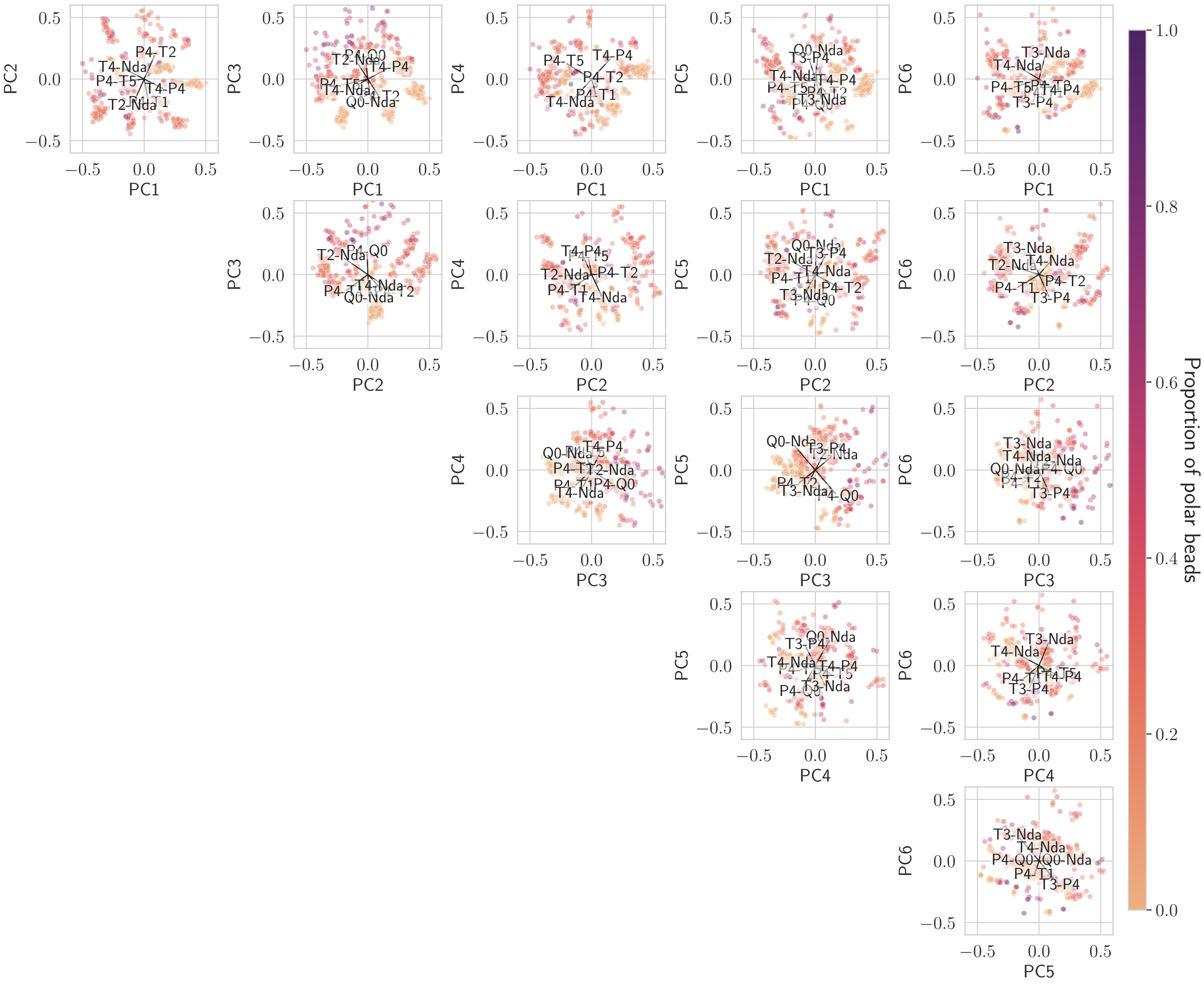

Figure S29: Biplots of all pairs of principal components with the largest six loadings corresponding to two-body interactions. The samples are colored by the ratio of polar beads (T1 and T2) per solute. We have found both PC3 an PC1 to be correlated to polarity (Figure S8), which again results in increased color separation in the respective biplot.

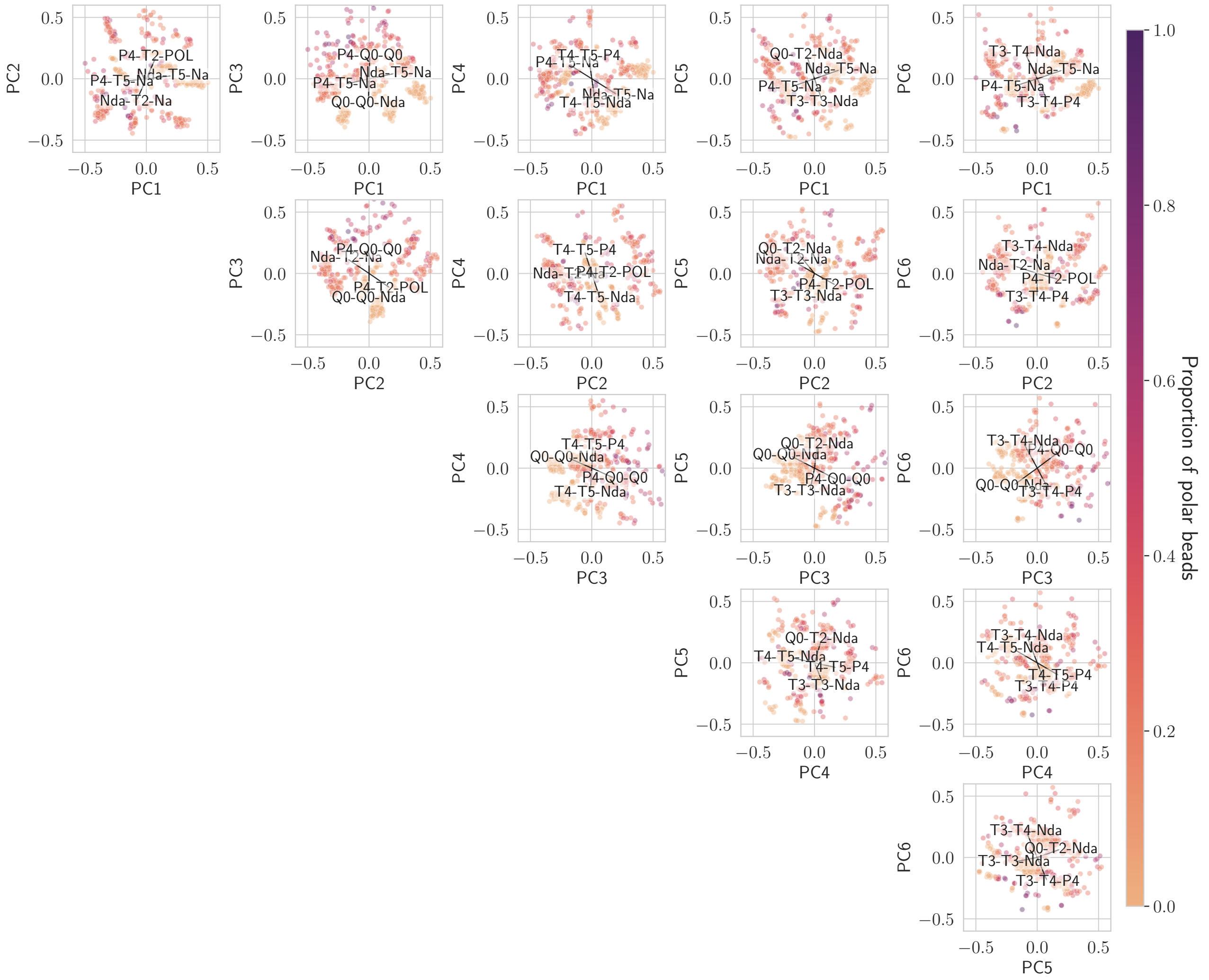

Figure S30: Biplots of all pairs of principal components with the largest four loadings corresponding to three-body interactions. The samples are colored by the ratio of polar beads (T1 and T2) per solute. We have found both PC3 an PC1 to be correlated to polarity (Figure S8), which again results in increased color separation in the respective biplot.

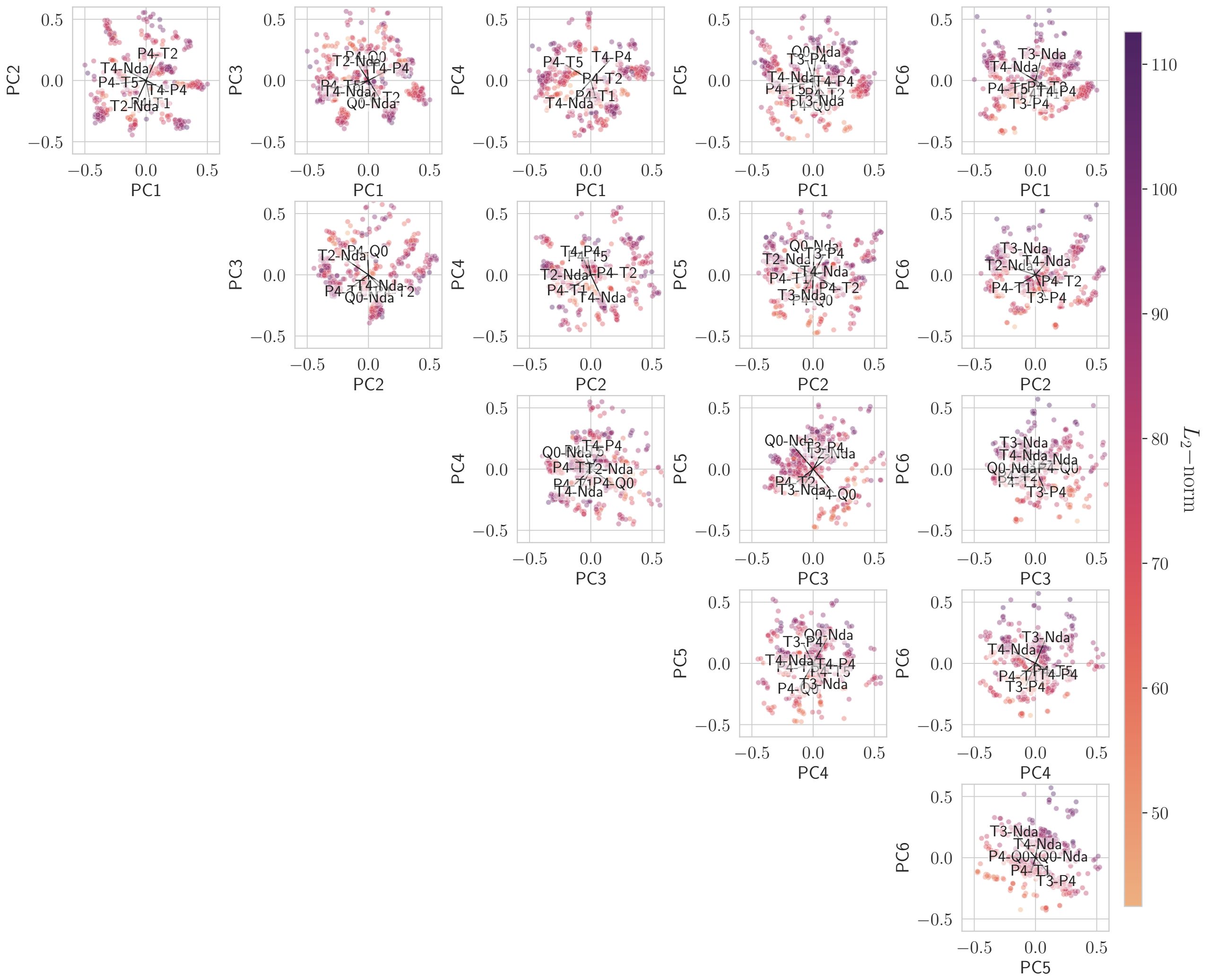

Figure S31: Biplots of all pairs of principal components with the largest six loadings corresponding to two-body interactions. The samples are colored by the difference in observed interactions, expressed as the L^2-norm (Figure S11). PC6 and PC5 are both correlated to the L^2-norm, leading to the most clear color separation in the corresponding plot.

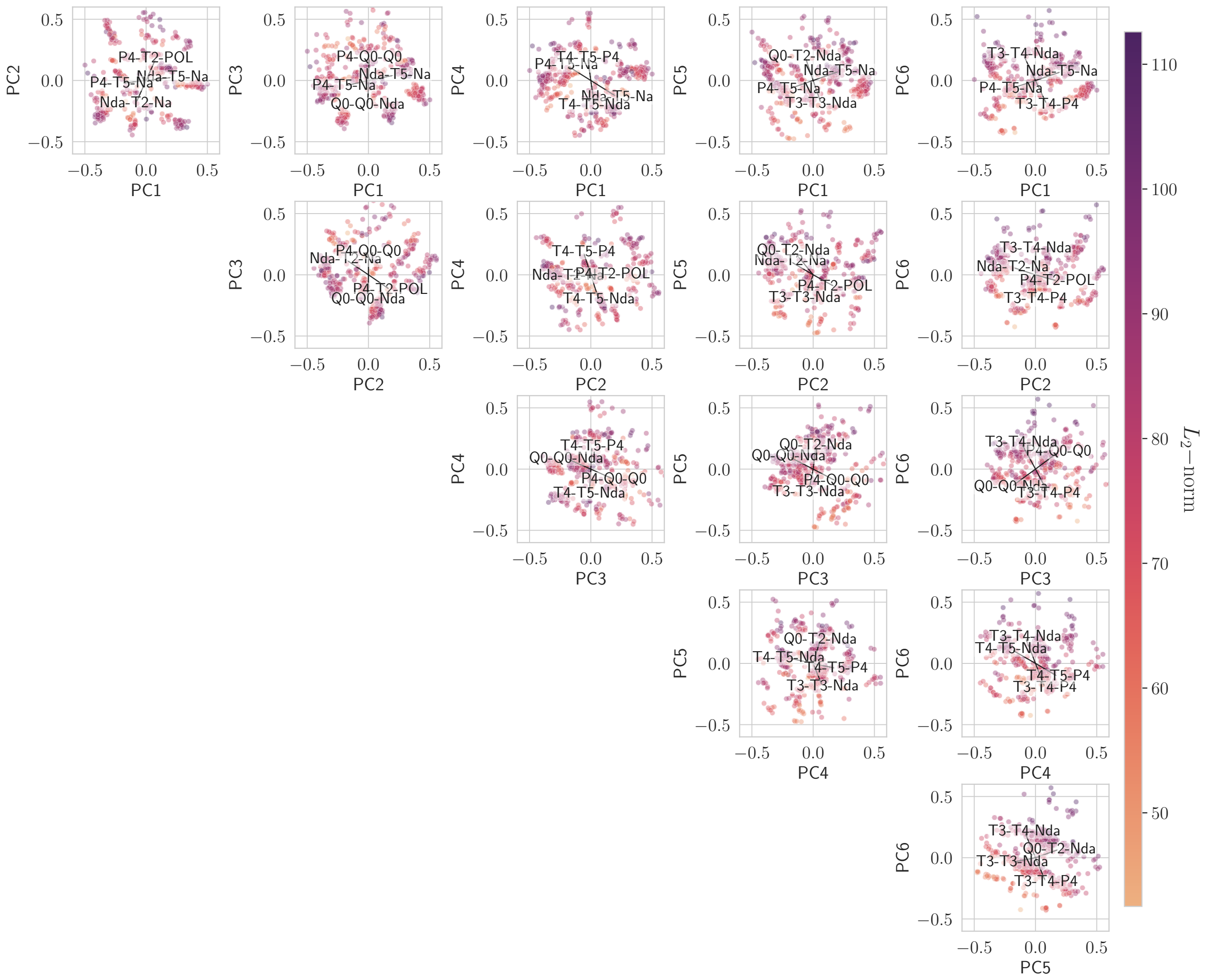

Figure S32: Biplots of all pairs of principal components with the largest four loadings corresponding to three-body interactions. The samples are colored by the difference in observed interactions, expressed as the L^2-norm (Figure S11). PC6 and PC5 are both correlated to the L^2-norm, leading to the most clear color separation in the corresponding plot.

References

[1] Bernadette Mohr, Kirill Shmilovich, Isabel S Kleinwächter, Dirk Schneider, Andrew L Ferguson, and Tristan Bereau. Data-driven discovery of cardiolipin-selective small molecules by computational active learning. *Chemical Science*, 13(16):4498–4511, 2022.

[2] Kiran H Kanekal and Tristan Bereau. Resolution limit of data-driven coarse-grained models spanning chemical space. *The Journal of chemical physics*, 151(16):164106, 2019.

[3] Siewert J Marrink, H Jelger Risselada, Serge Yefimov, D Peter Tieleman, and Alex H De Vries. The martini force field: coarse grained model for biomolecular simulations. *The Journal of Physical Chemistry B*, 111(27):7812–7824, 2007.

[4] Bernadette Mohr, Diego Van der Mast, and Tristan Bereau. STRUCTURAL_ANALYSIS, February 2023. URL `https://github.com/Bernadette-Mohr/STRUCTURAL_ANALYSIS`. Date accessed: 2023-02-01.

[5] AS Christensen, FA Faber, B Huang, LA Bratholm, A Tkatchenko, KR Muller, and OA von Lilienfeld. Qml: A python toolkit for quantum machine learning. *URL https://github. com/qmlcode/qml*, 2017. Date accessed: 2023-02-01.

[6] *Weighted Arithmetic Mean*, pages 565–566. Springer New York, New York, NY, 2008. ISBN 978-0-387-32833-1. doi: 10.1007/978-0-387-32833-1_421. URL `https://doi.org/10.1007/978-0-387-32833-1_421`.

[7] Marc Peter Deisenroth, A Aldo Faisal, and Cheng Soon Ong. *Mathematics for machine learning*. Cambridge University Press, 2020.

[8] Andrew L Ferguson. Machine learning and data science in soft materials engineering. *Journal of Physics: Condensed Matter*, 30(4):043002, 2017.

[9] Aldo Glielmo, Brooke E Husic, Alex Rodriguez, Cecilia Clementi, Frank Noé, and Alessandro Laio. Unsupervised learning methods for molecular simulation data. *Chemical Reviews*, 121(16): 9722–9758, 2021.

[10] Jörg Blasius, Paul HC Eilers, and John Gower. Better biplots. *Computational Statistics & Data Analysis*, 53(8):3145–3158, 2009.

Contents

1 Coarse-Grained Representations **1**
1.1 Calculation of solute water-octanol partitioning coefficients $\Delta G_{\mathrm{W}\rightarrow\mathrm{Ol}}$ 3

2 Determination of admissible three-body interactions by the SLATM representation **3**

3 Data Preprocessing **4**
3.1 Weighted arithmetic mean over many-body interaction spectra 4
3.2 Normalization of the Data 4
3.3 Selection of the Main Principal Components 4

4 Complete Set of Cross-correlations **6**

5 Correlations between the first six principal components and the many-body interactions **10**

6 Interaction graphs **13**

7 Biplots of the main six principal components and their corresponding eigenvectors 20

List of Figures

S1 Coarse-grained *5 + 1* model 1
S2 Illustration of the graph structures found in the analyzed samples. 2
S3 Distribution of the interaction frequencies in the SLATM representations. 5
S4 Difference vector $\Delta\langle\mathcal{X}\rangle$ between the mean log-normalized interaction frequencies in CL and PG. 5
S5 Scree Plot showing the amount of variance in the data set explained by the first 10 components identified by PCA. 6
S6 Cross-correlation of the first six principal components and the selectivity metric ($\Delta\Delta G$). 7
S7 Cross-correlation of the first six principal components and the average water-octanol partitioning free energy of the solutes $\Delta G_{\mathrm{W}\rightarrow\mathrm{Ol}}$. 7
S8 Cross-correlation of the first six principal components and the number of polar beads per solute. 8
S9 Cross-correlation of the first six principal components and the number of charged beads per solute. 8
S10 Cross-correlation of the first six principal components and the number of hydrogen-bonding beads per solute. 9
S11 Cross-correlation of the first six principal components and the Euclidian distance of the $\Delta\langle\mathcal{X}\rangle$. 9
S12 Scaled loadings of the many-body interactions of PC1, PC2 and PC3. 11
S13 Scaled loadings of the many-body interactions of PC4, PC5 and PC6. 12
S14 Illustration of the two- and three-body interactions used to analyze the structure–property relationship. 13
S15 Graph visualization of the two-body and three-body interactions with highest correlation to PC3. 14
S16 Graph visualization of the two-body and three-body interactions with highest correlation to PC1. 15
S17 Graph visualization of the two-body and three-body interactions with highest correlation to PC2. 16
S18 Graph visualization of the two-body and three-body interactions with highest correlation to PC4. 17
S19 Graph visualization of the most common interactions generated by analyzing the two-body and three-body interactions with the maximum absolute values of $l_{5,\mathrm{scaled}}$. 18
S20 Graph visualization of the two-body and three-body interactions with the maximum absolute values of $l_{6,\mathrm{scaled}}$. 19
S21 Biplots of all pairs of principal components, two-body interactions, ratio of charged beads per solute. 21
S22 Biplots of all pairs of principal components, three-body interactions, ratio of charged beads per solute. 22
S23 Biplots of all pairs of principal components, two-body interactions, average hydrophobicity $\Delta G_{\mathrm{W}\rightarrow\mathrm{Ol}}$. 23
S24 Biplots of all pairs of principal components, three-body interactions, average hydrophobicity $\Delta G_{\mathrm{W}\rightarrow\mathrm{Ol}}$. 24
S25 Biplots of all pairs of principal components, two-body interactions, selectivity $\Delta\Delta G$. . 25
S26 Biplots of all pairs of principal components, three-body interactions, selectivity $\Delta\Delta G$. 26
S27 Biplots of all pairs of principal components, two-body interactions, ratio of hydrogen-bonding beads per solute. 27
S28 Biplots of all pairs of principal components, three-body interactions, ratio of hydrogen-bonding beads per solute. 28

S29 Biplots of all pairs of principal components, two-body interactions, ratio of polar beads per solute. . . . 29
S30 Biplots of all pairs of principal components, three-body interactions, ratio of polar beads per solute. . . . 30
S31 Biplots of all pairs of principal components, two-body interactions, Euklidian distance. 31
S32 Biplots of all pairs of principal components, three-body interactions, Euklidian distance. 32
